# Different correlation of body mass index with body fatness and obesity-related biomarker according to age, sex and race-ethnicity

**DOI:** 10.1038/s41598-023-30527-w

**Published:** 2023-03-01

**Authors:** Su-Min Jeong, Dong Hoon Lee, Leandro F. M. Rezende, Edward L. Giovannucci

**Affiliations:** 1 Department of Medicine, Seoul National University College of Medicine, Seoul, Republic of Korea; 2 Department of Family Medicine, Seoul National University Health Service Center, Seoul, Republic of Korea; 3 Department of Family Medicine, Seoul National University Hospital, Seoul, Republic of Korea; 4 Department of Sport Industry Studies, Yonsei University, Seoul, Republic of Korea; 5 Department of Nutrition, Harvard T.H. Chan School of Public Health, Boston, MA 02115 USA; 6 Department of Preventive Medicine, Escola Paulista de Medicina, Universidade Federal de São Paulo, Sao Paulo, SP Brazil; 7 Department of Epidemiology, Harvard T.H. Chan School of Public Health, 665 Huntington Avenue, Bldg. 2, Room 371, Boston, MA 02115 USA

**Keywords:** Epidemiology, Nutrition

## Abstract

The relationship between body mass index (BMI) and body fatness could differ according to age, sex, and race-ethnicity. We aimed to evaluate in which contexts BMI could be a good measure for body fatness compared to dual-energy X-ray absorptiometry (DXA) derived measures. The study population included 18,061 participants (9141 men and 8920 women) aged 18 and older who tested DXA from the National Health and Nutrition Examination Survey (NHANES) database from 1999 to 2006, and 8107 men and 10,754 women with DXA data from Korea NHANES from 2008 to 2011 to represent the Asian population. We calculated Pearson correlation coefficients between BMI and DXA derived fat mass index (FMI) and percentage body fat (PBF) depending on age, sex, and race-ethnicity. The correlation between BMI, FMI and PBF and obesity-related biomarkers was also estimated among the subgroup with both DXA and information on each biomarker. BMI was strongly correlated with FMI (r = 0.944 in men and 0.976 in women), PBF (r = 0.735 in men and 0.799 in women), and truncal fat mass (r = 0.914 in men and 0.941 in women) with correlations stronger in women than in men except for with waist-height ratio (r = 0.921 in men and 0.911 in women). The correlation between BMI and DXA derived adiposity weakened with age in both sexes. BMI was less correlated with FMI (r = 0.840 in men and 0.912 in women), PBF (r = 0.645 in men and 0.681 in women), and truncal fat mass (r = 0.836 in men and 0.884 in women) in Korean compared to other race-ethnicities. Among obesity-related biomarkers, insulin was the most strongly correlated to body adiposity indices in both sexes and strength of these correlations generally decreased with age. BMI predicted obesity-related biomarkers as well as FMI and truncal fat mass and superior to PBF. BMI could be a good measure for body fatness, particularly among young age groups, women, the US population, but less so in Korean populations. The lower correlation between BMI and body fatness in older compared to younger age groups could be related to increasing PBF and decreasing lean body mass.

## Introduction

Body mass index (BMI) is a widely used indicator for adiposity because it is simple and inexpensive to measure. However, BMI does not differentiate between fat mass and lean mass. BMI is generally well correlated to percentage body fat, with correlations ranging from 0.72 to 0.86^1–5^, but this correlation varies depending on age, sex or race/ethnic groups. A study for 23,627 UK adults reported that age independently affected percentage body fat^6^. Percentage body fat increased with age at a fixed BMI due to steady increase in fat mass and slight decrease in lean mass with increasing age. In one study, BMI and waist circumference (WC) were well correlated with percentage body fat, showing decreased trends of correlation coefficients as participants increased in age^1^. For example, men aged 20–39 years had a higher correlation between BMI and percentage body fat (r = 0.789) than men aged more than 80 years or more (r = 0.716). Similarly, a study of 202 Black and 504 White adults found that older people had a higher percentage body fat compared with younger people for the same BMI regardless of ethnicity^7^. Meanwhile, the relationship between BMI and percentage body fat is also different according to race/ethnic groups. Asians have a lower BMI but a higher percentage body fat compared to age-matched Whites^8^. Blacks generally have a greater bone mineral density, body protein and lean mass than Whites, which results in a racial difference in the relationship between BMI and percentage body fat^9^.

Abdominal obesity reflected by WC and truncal fat also had different patterns according to age, sex, and ethnicity^10^. There was more truncal fat with increasing age in men and women across all ethnicities, but its relationship with BMI dependent on age, sex, and ethnicity is less well understood. Asian ethnic groups have lower values of WC compared with Whites, but lower WC cutoff for cardiometabolic risk factors than Whites as WC correlates with body frame size and height^11^. In this context, body height was highlighted as an important co-factor for body fatness indices^12^. Body height has been negatively associated with BMI, suggesting shortness is related to having a higher BMI^13^. Waist-to-height ratio (WHtR) has been proposed as a more sensitive adiposity index for central obesity as well as superior predictor of cardiovascular risk than BMI^14^.

Furthermore, body fat indices have correlations with cardiometabolic biomarkers, resulting in comorbidities of obesity. Excess body fat is an independent risk factor for several comorbidities of obesity such as diabetes mellitus, coronary heart diseases and some cancers, resulting from insulin resistance, endothelial dysfunction, and altered lipid metabolism^15^. Although a higher BMI itself was associated with insulin resistance and metabolic syndrome^16^, percentage body fat could be more closely related to cardiovascular risk than BMI^17,18^. Moreover, normal BMI with high percentage body fat had a four-fold higher prevalence of metabolic syndrome compared with normal BMI with low percentage body fat^17^. Recent studies suggested that examining body composition may be superior to BMI in examining health outcomes^19,20^. However, the relationship between body adiposity and cardiometabolic biomarkers with respect to age, sex, and ethnic group has not been fully examined.

As Asian Americans are a growing subpopulation in the US making up 6.2% of the US population in 2020, the National Health and Nutrition Examination Survey (NHANES) started to identify Asian Americans separated from other ethnic groups since 2011^21^. Before 2011, the Asian population was included into ‘other’ ethnic group without specific categorization in the US NHANES data. Thus, we added Korean population from Korea NHANES, which contains information of body composition measured with same methods as in the US NHANES^22^. The Korean population was estimated as 1.5 million people in the US and ranked as fifth largest Asian population in the US^23^.

Therefore, the aim of this study was to evaluate in which context BMI reflects more accurately body fatness as intended according to age, sex, race/ethnic groups, and presence of chronic diseases using nationally representative population samples from US and South Korea. In addition, we sought to investigate how well body fatness indices, especially fat mass, and percentage of body fat compared with BMI correlate with obesity-related biomarkers and how these correlations change according to sex and age.

## Methods

### Study setting and study populations

The National Health and Nutrition Examination Survey (NHANES) is a series of cross-sectional surveys of the US civilian, noninstitutionalized population selected by a complex, multistage probability sampling design. We used data from 1999 to 2006 so that we can use information on body composition assessed by dual-energy X-ray absorptiometry (DXA). The study population initially included 20,378 participants older than 18 years old and who had information on DXA measurements. After excluding those who had highly variable imputed DXA data due to missing information on weight and WC or who did not have imputed data because of pregnancy (n = 1593), and those who had no anthropometric measures (n = 724), we finally included 18,061 participants (9141 men and 8920 women). For Korea NHANES (KNHANES), we included the 18,861 participants (8107 men and 10,754 women) older than 18 years old and who had undergone DXA exam from 2008 to 2011 after excluding missing data for DXA, BMI, and WC (n = 679). In both dataset, subsets consisting of participants who have information on each biomarker were used when we conducted correlation analyses between body composition and obesity-related biomarkers. (Supplementary Fig. 1 and Supplementary Fig. 2) The NHANES and KNHANES data are publicly available without personal identifiable information and were exempt of ethics approval as a secondary analysis of public data.

### Measurements of anthropometry and body composition

Anthropometric measures such as height (cm), weight (kg), and WC (cm) were assessed by trained technicians with standardized protocols. WC was measured at the uppermost lateral border of iliac crest. BMI was calculated as weight (kg) divided by height (m) squared and categorized into four groups (< 18.5, 18.5–24.9, 25–29.9, ≥ 30 kg/m^2^) according to World Health Organization criteria^24^.

Whole-body DXA exams were conducted at the Mobile Examination Center using a Hologic QDR 4500A fan beam X-ray bone densitometer (Hologic Inc., Waltham, MA, USA)^25^. The DXA scans were reviewed for quality control and analyzed using Hologic Discovery software (version 12.1). Data for total body fat mass, total body lean mass and percentage body fat was used in our study. To correct for the effect of body size, fat mass and lean mass were respectively converted to fat mass index (fat mass [kg] divided by height [m^2^]) and lean mass index (lean mass [kg] divided by height [m^2^]) considering that fat mass and lean mass are largely determined by height^26^. Truncal region was defined by DXA includes subcutaneous and intermuscular fat of neck, trunk and pelvis. Appendicular skeletal muscle index was calculated as the sum of skeletal muscle in the arms and legs measured by DXA scans divided by height (m) squared, equivalent to all non-fat and non-bone tissue. To examine the different correlations by race/ethnicity groups, DXA data from KNHANES was supplemented since the NHANES data set did not separately classify and include a sizable enough Asian ethnic population. In the KNHANES, whole-body DXA exams were conducted with the same equipment as in NHANES (Hologic QDR 4500A) and analyzed with industry standard techniques using Hologic Discovery software (version13.1) in its default configuration.

### Other covariates

Age was categorized as 18–29, 30–39, 40–49, 50–59, 60–69, and ≥ 70 years. Race/ethnicity were classified as White, Black, Mexican–American, other Hispanic, and others, and Korean. For racial differences, others from NHANES were excluded because we could not identify specific race/ethnicity due to lack of information. Smoking status was categorized into never, past and current based on self-reported questionnaire. Presence of chronic diseases was defined when participants have as least one of the following chronic diseases: cancer, coronary heart disease, congestive heart failure, stroke, thyroid disease, chronic pulmonary disease, and liver disease. Total cholesterol (TC), low-density lipoprotein cholesterol (LDL-C), high-density lipoprotein cholesterol (HDL-C), and triglycerides, C-reactive protein (CRP), serum glucose levels and insulin were measured using standard techniques.

### Statistical analysis

Analyses were performed using SAS version 9.4 (SAS Institute INC.), SAS-callable SUDAAN (Research Triangle Institute) and all analyses account for the sample weights and multiple imputations. DXA Multiple Imputation Data Files in which missing and invalid DXA scans were imputed five times using multiple imputation technique was used as released by the National Center for Health Statistics^27^. Older and obese people tend to be more likely to have missing data on DXA in NHANES; multiple imputation of the missing DXA was provided^28^. We estimated sample-weighted Pearson correlation coefficients between body composition variables (fat mass index, percentage body fat, lean mass index, truncal fat mass, and appendicular skeletal muscle index) and BMI within each age-sex group. We also examined the weighted correlations between obesity-related biomarkers (e.g., cholesterol, CRP, and insulin) and body measures (BMI, fat mass index, and percentage body fat). Partial Pearson correlation controlling for age was obtained to see relationship between BMI and DXA derived body measured by chronic diseases. Student’s t test for continuous variables and Chi square test for categorical variables were performed to compare baseline characteristic between men and women.

### Ethics approval

For each survey (NHANES and Korea NHANES), the relevant institutional or national ethics review board approved the survey. All participants provided written informed consent, and the institutional review board (IRB) of the US Centers for Disease Control and Prevention and Korea Centers for Disease Control and Prevention (KCDC) approved the study (IRB: Protocol #98-12 and Protocol #2005-06 for NHANES 2008-04EXP-01-C, 2009-01CON-03-2C, 2010-02CON-21-C, and 2011-02CON-06-C for Korea NHANES).

## Results

### Characteristics of study population

Supplementary Table 1 shows descriptive data for the study population stratified by sex. The average age was 43.2 (standard error [SE] 0.3) for men and 45.3 (SE 0.3) for women. In KNHANES, the average age was 43.2 (SE 0.3) for men and 45.5 (SE 0.3) for women. The distribution of participants according to age category was 21.8% (18–29 years), 20.1% (30–39 years), 22.1% (40–49 years), 17.0% (50–59 years), 10.8%, (60–69 years) and 8.2% (≥ 70 years). The mean BMI was higher in women than in men (28.2 vs. 27.9 kg/m^2^). A higher prevalence of abdominal obesity based on the sex-specific criteria was observed in women than in men (57.8% vs. 39.5%). Men had 5.5 kg lower fat mass and 17.7 kg higher lean mass leading to 11.9% lower percentage body fat compared with women. The majority of the participants were Whites (71.1%). In terms of biomarkers, men had higher LDL-C, lower HDL-C, and lower insulin level compared with women. The Korean population distribution by age group was 21.0% (18–29 years), 21.2% (30–39 years), 21.9% (40–49 years), 17.1% (50–59 years), 10.2% (60–69 years) and 8.6% (≥ 70 years). Korean men had higher BMI (24.0 vs. 23.1 kg/m^2^), WC (83.7 vs. 77.6 cm), and lower percentage body fat (22.0 vs. 32.9%) than Korean women.

Comparisons between included vs. excluded population is shown in Supplementary Table 2. The included population tended to be less obese than excluded population in NHANES.

### Correlations between BMI and DXA-derived indices by age, sex and race-ethnicity

In Table 1, average values of body measures and Pearson correlations between BMI and DXA-derived indices (fat mass index, percentage body fat, and lean mass index) by age and sex are shown for each stratum. BMI displayed linear relations with fat mass index and curvilinear relationships with percentage body in all sex and age groups (Supplementary Fig. 3 and Supplementary Fig. 4). Fat mass index had a stronger correlation with BMI than percentage body fat in all sex and age groups. The correlations between BMI and fat mass index, percentage body fat, lean mass index, and appendicular skeletal muscle index were generally high, but were slightly higher in women than for men. Overall, BMI, WC and fat mass index tended to increase as age increased until 60–69 years of age for both men and women, and then slightly slope downward. WHtR became higher with increasing age. Lean mass index also increased with maximal point of 19.7 kg/m^2^ in men aged 40–49 years and 16.3 kg/m^2^ in women aged 40–49 years and has the lowest values in both men and women aged 70 years or older (17.9 kg/m^2^ for men and 15.2 kg/m^2^ for women). Meanwhile, percentage body fat showed an increasing trend as age increased in both men and women.

**Table 1:** Pearson correlation of waist circumference, percentage body fat, lean body mass index and fat mass index with body mass index by sex and age group in National Health and Nutrition Examination Survey (NHANES) and Korea NHANES.

Age group (years)	BMI, kg/m^2^	WC, cm	Height, cm	WHtR	FMI, kg/m^2^	PBF, %	TF, kg	LMI, kg/m^2^	ASMI, kg/m^2^	WC	Height	WHtR	FMI	PBF	TF	LMI	ASMI
			Mean (SE)							Correlation coefficients with BMI							
NHANES																	
Men	27.9 (0.1)	99.3 (0.3)	176.3 (0.1)	0.56	8.1 (0.1)	27.7 (0.1)	13.1 (0.1)	19.2 (0.1)	8.6 (0.02)	0.923	0.031	0.921	0.944	0.765	0.914	0.918	0.821
18–29	26.4 (0.1)	92.0 (0.4)	177.1 (0.2)	0.52	7.0 (0.1)	24.9 (0.2)	10.4 (0.2)	18.8 (0.1)	8.6 (0.04)	0.946	0.067	0.952	0.952	0.821	0.932	0.928	0.866
30–39	27.8 (0.2)	97.3 (0.5)	176.5 (0.3)	0.55	7.8 (0.1)	26.7 (0.2)	12.4 (0.2)	19.5 (0.1)	8.8 (0.04)	0.937	0.065	0.940	0.945	0.764	0.921	0.927	0.858
40–49	28.8 (0.2)	101.6 (0.5)	177.2 (0.2)	0.57	8.4 (0.1)	28.2 (0.2)	14.0 (0.2)	19.7 (0.1)	8.8 (0.04)	0.934	− 0.009*	0.943	0.954	0.770	0.926	0.938	0.862
50–59	28.7 (0.2)	103.4 (0.5)	176.3 (0.3)	0.59	8.7 (0.1)	29.3 (0.2)	14.7 (0.2)	19.4 (0.1)	8.5 (0.04)	0.920	− 0.023*	0.937	0.943	0.758	0.902	0.923	0.835
60–69	29.0 (0.2)	105.9 (0.5)	175.1 (0.3)	0.61	9.1 (0.1)	30.6 (0.2)	15.5 (0.2)	19.2 (0.1)	8.3 (0.04)	0.931	0.050*	0.936	0.940	0.739	0.908	0.915	0.823
≥ 70	27.3 (0.1)	102.8 (0.4)	172.3 (0.2)	0.60	8.7 (0.1)	31.0 (0.2)	13.8 (0.2)	17.9 (0.1)	7.6 (0.03)	0.916	0.061	0.912	0.932	0.736	0.891	0.892	0.795
Women	28.2 (0.1)	93.0 (0.3)	162.1 (0.1)	0.57	11.7 (0.1)	39.6 (0.1)	14.5 (0.1)	15.9 (0.1)	6.7 (0.02)	0.911	− 0.044	0.911	0.976	0.799	0.941	0.926	0.877
18–29	26.3 (0.2)	87.1 (0.5)	163.2 (0.2)	0.53	10.2 (0.1)	36.7 (0.2)	12.1 (0.2)	15.6 (0.1)	6.7 (0.03)	0.928	− 0.012*	0.936	0.979	0.838	0.954	0.930	0.892
30–39	27.9 (0.3)	90.7 (0.6)	163.1 (0.2)	0.56	11.3 (0.2)	38.4 (0.3)	13.9 (0.2)	16.1 (0.1)	6.9 (0.04)	0.929	− 0.064	0.934	0.979	0.824	0.953	0.932	0.892
40–49	28.8 (0.3)	93.9 (0.6)	163.3 (0.2)	0.58	11.9 (0.2)	39.6 (0.2)	15.1 (0.2)	16.3 (0.1)	6.9 (0.04)	0.918	− 0.058	0.930	0.979	0.808	0.943	0.938	0.907
50–59	29.4 (0.3)	96.3 (0.7)	162.2 (0.2)	0.59	12.6 (0.2)	41.4 (0.2)	16.3 (0.2)	16.2 (0.1)	6.7 (0.04)	0.911	− 0.063	0.917	0.979	0.790	0.940	0.935	0.902
60–69	29.5 (0.2)	98.4 (0.5)	161.3 (0.2)	0.61	12.9 (0.2)	42.6 (0.2)	16.5 (0.2)	16.0 (0.1)	6.6 (0.04)	0.893	− 0.020*	0.899	0.976	0.782	0.922	0.927	0.892
≥ 70	27.4 (0.2)	95.5 (0.4)	158.0 (0.2)	0.60	11.7 (0.1)	41.5 (0.2)	14.1 (0.2)	15.2 (0.1)	6.2 (0.04)	0.871	− 0.036*	0.874	0.965	0.751	0.884	0.893	0.841
Korea NHANES																	
Men	24.0 (0.1)	83.7 (0.2)	170.7 (0.1)	0.49	5.3 (0.03)	22.0 (0.1)	8.4 (0.1)	17.5 (0.03)	7.7 (0.02)	0.869	0.016*	0.836	0.840	0.645	0.836	0.846	0.755
18–29	23.4 (0.1)	80.2 (0.3)	174.5 (0.2)	0.46	5.0 (0.1)	21.0 (0.2)	7.6 (0.1)	17.3 (0.1)	7.8 (0.03)	0.911	− 0.026*	0.925	0.877	0.717	0.878	0.851	0.781
30–39	24.3 (0.1)	83.7 (0.3)	172.9 (0.2)	0.48	55 (0.1)	22.3 (0.2)	8.9 (0.1)	17.7 (0.1)	7.9 (0.03)	0.894	0.006*	0.901	0.856	0.667	0.843	0.842	0.773
40–49	24.4 (0.1)	84.8 (0.3)	170.5 (0.2)	0.50	5.4 0.1)	22.0 (0.2)	8.8 (0.1)	17.8 (0.1)	7.8 (0.03)	0.879	0.023*	0.884	0.827	0.617	0.829	0.850	0.768
50–59	24.2 (0.1)	85.5 (0.3)	168.5 (0.2)	0.51	5.3 (0.1)	22.0 (0.2)	8.6 (0.1)	17.8 (0.1)	7.7 (0.03)	0.836	0.004*	0.846	0.775	0.534	0.761	0.839	0.761
60–69	23.8 (0.1)	85.9 (0.3)	166.3 (0.2)	0.52	5.4 (0.1)	22.5 (0.2)	8.5 (0.1)	17.3 (0.1)	7.4 (0.03)	0.841	0.015*	0.850	0.832	0.648	0.807	0.830	0.729
≥ 70	22.7 (0.1)	84.0 (0.4)	164.1 (0.2)	0.51	5.3 (0.1)	23.0 (0.2)	8.0 (0.1)	16.4 (0.1)	7.0 (0.03)	0.888	0.117*	0.880	0.850	0.662	0.837	0.840	0.719
Women	23.1 (0.1)	77.6 (0.2)	157.2 (0.1)	0.50	7.7 (0.1)	32.9 (0.1)	9.4 (0.1)	14.5 (0.03)	5.9 (0.01)	0.863	− 0.190	0.844	0.912	0.681	0.884	0.838	0.744
18–29	21.4 (0.1)	71.2 (0.3)	161.5 (0.2)	0.44	6.9 (0.1)	31.6 (0.2)	8.0 (0.1)	13.6 (0.1)	5.6 (0.03)	0.857	− 0.046*	0.878	0.927	0.709	0.908	0.850	0.794
30–39	22.5 (0.1)	75.0 (0.3)	159.7 (0.1)	0.47	7.2 (0.1)	31.9 (0.2)	8.8 (0.1)	14.2 (0.1)	5.8 (0.03)	0.873	− 0.117	0.894	0.918	0.677	0.897	0.856	0.795
40–49	23.5 (0.1)	77.7 (0.3)	157.9 (0.1)	0.49	7.7 (0.1)	32.6 (0.2)	9.6 (0.1)	14.7 (0.1)	6.0 (0.02)	0.859	− 0.098	0.868	0.901	0.653	0.880	0.832	0.756
50–59	24.2 (0.1)	81.1 (0.3)	155.6 (0.2)	0.52	8.3 (0.1)	34.2 (0.2)	10.6 (0.1)	14.9 (0.1)	6.0 (0.02)	0.840	− 0.101	0.849	0.868	0.579	0.820	0.787	0.686
60–69	24.6 (0.1)	83.9 (0.3)	153.5 (0.2)	0.55	8.6 (0.1)	34.8 (0.2)	11.1 (0.1)	15.1 (0.1)	6.0 (0.02)	0.829	− 0.053	0.841	0.890	0.638	0.829	0.778	0.650
≥ 70	23.8 (0.1)	82.7 (0.3)	149.3 (0.2)	0.55	8.2 (0.1)	33.9 (0.2)	10.0 (0.1)	14.8 (0.1)	5.8 (0.02)	0.865	0.073	0.855	0.919	0.736	0.860	0.769	0.610

WC, waist circumference; FMI, fat mass index; PBF, percentage body fat; TF, truncal fat mass; LMI, lean mass index; ASMI, appendicular skeletal muscle index; BMI, body mass index; NHANES, National Health and Nutrition Examination Survey.

All *p* values < 0.05 except correlation between BMI and height with asterisk (*).

The degree of the correlations between fat mass index and BMI tended to slightly decrease with age in the US population, in particular participants aged ≥ 60 years for both men and women showing the lowest correlation, yet still high, in those aged ≥ 70 years (r = 0.932 in men and r = 0.965 in women) (Table 1). The degree of correlation between percentage body fat and BMI also decreased with age. In terms of fat distribution, the correlation between BMI and measures of abdominal obesity (WC, truncal fat mass, and WHtR) decreased with age.

**Figure 1:**
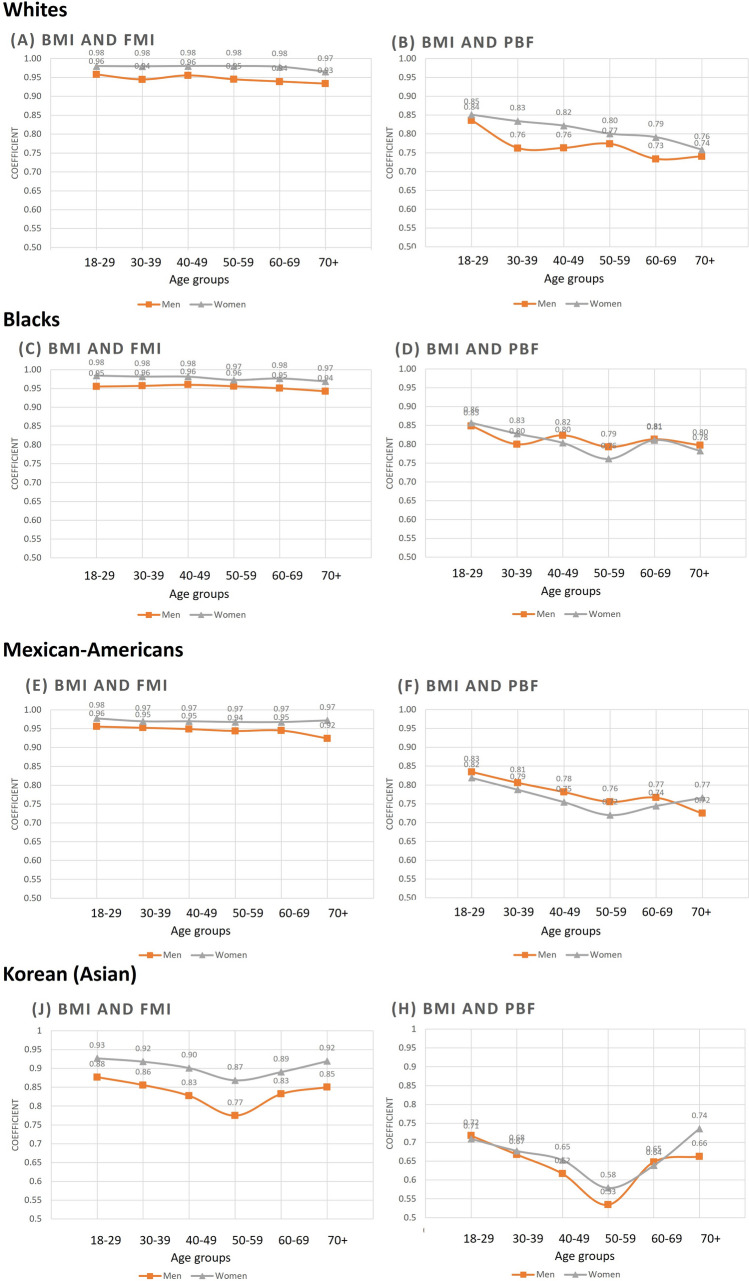
Pearson correlation coefficients between body mass index and DXA derived body adiposity (fat mass index and percentage body fat) by sex and age for each race-ethnicity. The bar graphs of (**A**–**H**) show coefficients between body mass index (BMI) and fat mass index (FMI) and percentage body fat (PBF) in (**A**) and (**B**) Whites; (**C**) and (**D**) Blacks; (**E**) and (**F**) Mexican-Americans, and (**G**) and (**H**) Koreans.

Women had higher correlations between DXA-derived adiposity (fat mass index and percentage body fat) and BMI compared with men across all different race/ethnicities (Fig. 1). Higher correlation was observed in Whites, Blacks, and Mexican-Americans than in Koreans (Supplementary Fig. 5 and Supplementary Fig. 6). The degree of correlation between fat mass index, percentage body fat, and truncal fat mass and BMI in Koreans showed different pattern by age; there was a nadir in group aged 50–59 years and upward slope (Table 1 and Fig. 1). This contrasted to the generally more linear decreasing pattern in the other racial/ethnic groups.

Men had slightly higher correlations between WHtR and BMI compared with women (r = 0.921 in men and r = 0.911 in women) unlike other DXA-derived adiposity measures (Table 1). The US population had a stronger correlation of WHtR with BMI than Korean population.

### Correlations between WHtR and DXA-derived indices by age and sex

Supplementary Table 3 shows Pearson correlation between WHtR and DXA-derived indices. In contrast to correlation of fat mass index and percentage body fat with BMI being stronger in women than men, the correlation between fat mass index and percentage body fat with WHtR was stronger in men than women. The degree of correlation of fat mass index and percentage body fat with WHtR decreased with age in NHANES, while Koreans had the lowest correlation in group with age 50–59 year. The correlation between WHtR and percentage body fat was generally stronger than correlation between BMI and percentage body fat.

### Correlations between BMI and DXA-derived indices by chronic diseases

Overall, BMI, adiposity indices, and lean mass index tended to be higher in groups with chronic diseases, except that lean mass index was lower in patients with cancer history (Supplementary Table 4). According to presence of chronic diseases, the correlation between BMI and percentage body fat/fat mass index appeared similar in both groups.

### Correlation of measures of adiposity with biomarkers

**Figure 2:**
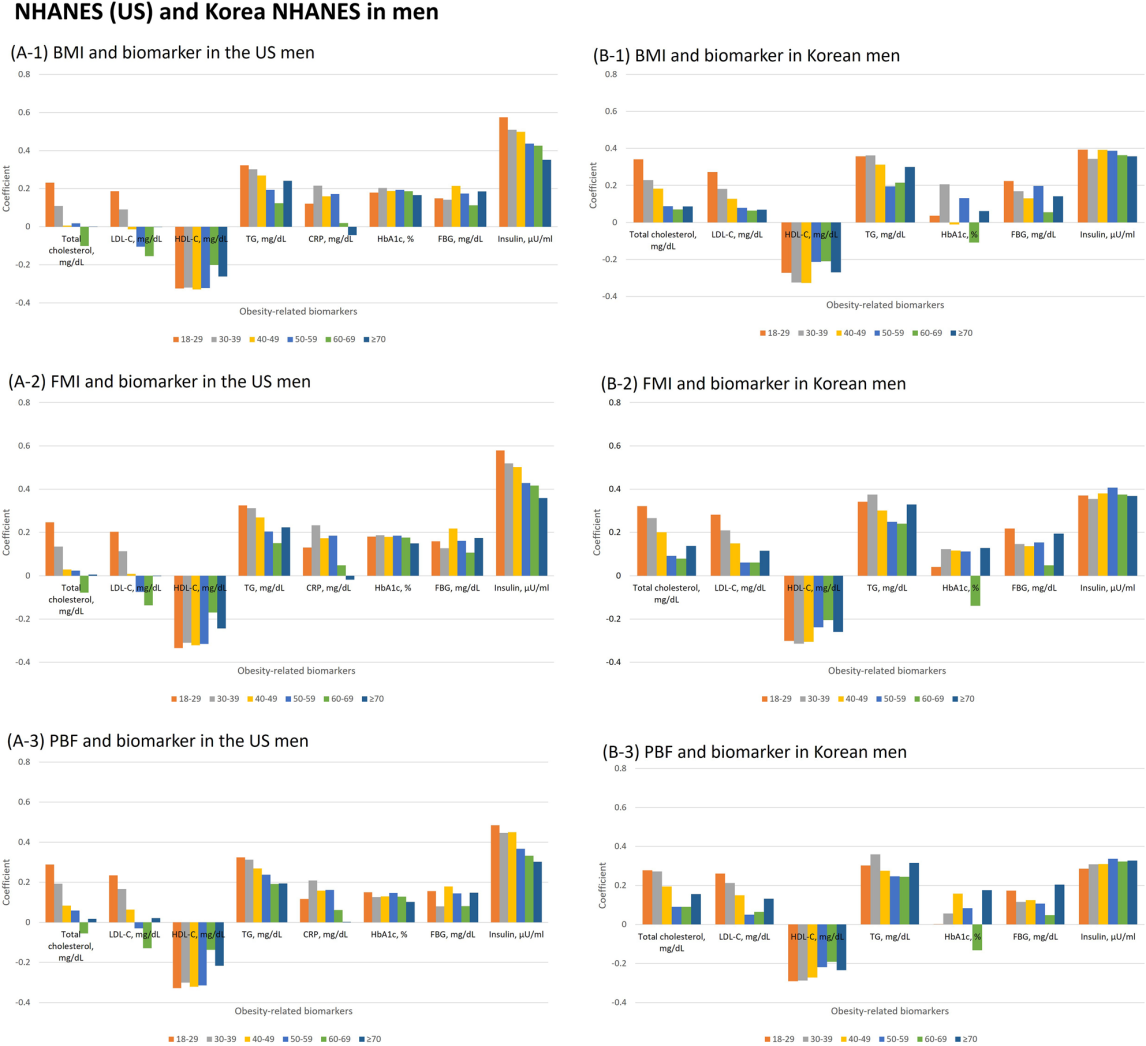
Pearson correlation coefficients between obesity-related biomarkers and body adiposity indices (body mass index, fat mass index and percentage body fat) by age in men. The bar graphs of panel A show coefficients between obesity-related biomarkers (total cholesterol, low-density lipoprotein cholesterol [LDL-C], high-density lipoprotein cholesterol [HDL-C], triglycerides [TG], C-reactive protein [CRP], hemoglobinA1c [HbA1c], fasting blood glucose [FBG] and insulin) and body mass index (BMI), fat mass index (FMI), and percentage body fat (PBF) in National Health and Nutrition Examination Survey (NHANES) and panel B shows the correlations in Korea NHANES. CRP was not available in Korea NHANES.

**Figure 3:**
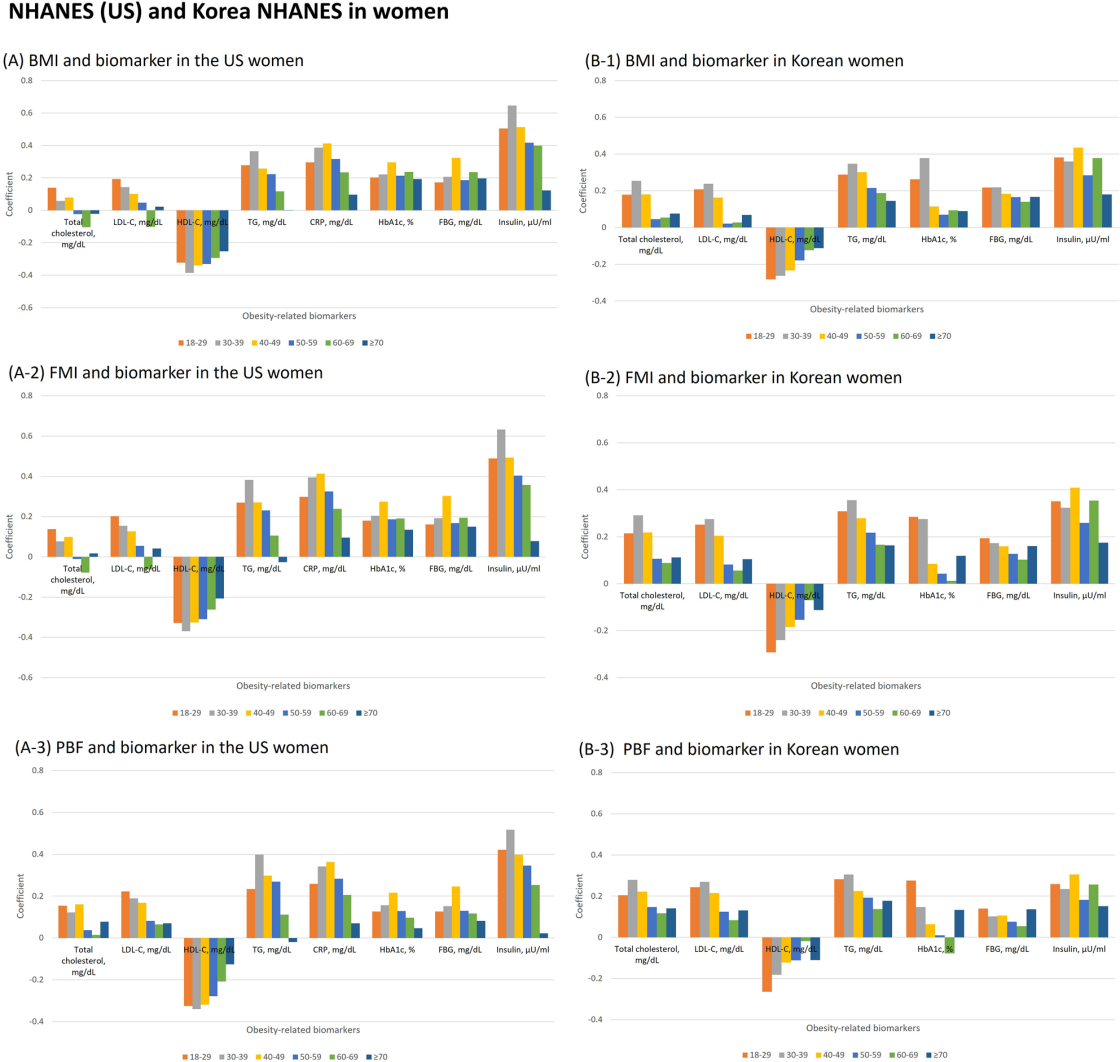
Pearson correlation coefficients between obesity-related biomarkers and body adiposity indices (body mass index, fat mass index and percentage body fat) by age in women. The bar graphs of panel A show coefficients between obesity-related biomarkers (total cholesterol, low-density lipoprotein cholesterol [LDL-C], high-density lipoprotein cholesterol [HDL-C], triglycerides [TG], C-reactive protein [CRP], hemoglobinA1c [HbA1c], fasting blood glucose [FBG] and insulin) and body mass index (BMI), fat mass index (FMI), and percentage body fat (PBF) in National Health and Nutrition Examination Survey (NHANES) and panel B shows the correlations in Korea NHANES. CRP was not available in Korea NHANES.

The correlation of measures of adiposity (BMI, fat mass index, and percentage body fat) with obesity-associated biomarkers by age was shown in men (Fig. 2) and in women (Fig. 3). BMI, fat mass index and percentage body fat were significantly correlated with each biomarker in both men (r ≤ 0.491) and women (r ≤ 0.647). In general, the correlations were comparable for BMI, fat mass index, and percentage body fat with the biomarkers, and none was clearly superior. Among biomarkers, insulin had the strongest correlation with measures of adiposity for both sexes. Correlation of insulin and fat mass index became linearly weaker with age in men (e.g., r = 0.575 in men aged 18–29 years and 0.351 in men aged ≥ 70 years).

In Koreans, the patterns were similar to NHANES. The correlations between the anthropometric measures and the biomarkers tended to decrease with age in all groups, except for insulin in Korean men, which had similar correlations across all age groups. In particular, the correlation with insulin was not strong in young aged Korean men in contrast to having the strongest correlation in NHANES. Measures of abdominal fat showed similar patterns in which the correlation between WC/truncal fat mass and insulin became linearly weaker with age in men (Supplementary Fig. 7). Difference in area under the curve (AUC) for identifying insulin resistance with Homeostasis Model Assessment of Insulin Resistance (cutoff points ≥ 3) across measures of adiposity was not significant (Supplementary Fig. 8). In the US population, the AUC among men was higher than women, while the AUC among Korean women was higher than men.

## Discussion

This study evaluated the extent to which the degree of correlations between BMI and DXA-derived body adiposity differed according to sex, age groups, race/ethnicity, and the presence of chronic diseases. In addition, the correlation between body adiposity measures and obesity-related biomarkers were examined according to sex and age. Our study showed that BMI reflects fat mass index and percentage body fat better in women than men, in younger age groups than older age groups, and in Whites/Blacks/Mexican–Americans than in Koreans (Asians). Among obesity-related biomarkers, insulin was most strongly correlated with BMI, fat mass index, and percentage body fat, and showed decreasing correlations with age. BMI and fat mass index were similarly correlated with biomarkers, and were slightly better than percentage body fat.

Previous studies have assessed the relationship of BMI with DXA-derived adiposity in various settings^29–31^. Overall, fat mass was more strongly correlated with BMI than with percentage body fat, consistent with our results. We found percentage body fat displayed a curvilinear relationship with BMI, where the slope between percentage body fat on BMI is steeper at lower BMI values. A previous study of 665 Black and White people also reported the relationship between BMI and percentage body fat was quadratic in individuals with BMI ≥ 35 kg/m^2^^32^. Negative regression coefficient of a quadratic term for BMI in our results supports curvilinear relationship between BMI and percentage body fat (data is not shown, *p* value < 0.001). When comparing men and women, the correlation between BMI and DXA-derived adiposity was stronger in women than men. Women have relatively more fat mass compared to men. This sex difference has been found even in childhood with girls aged 5–9 who had a greater fat mass than boys^29^. BMI was more strongly correlated with WC in men than in women, which is partly explained by different patterns of fat distribution. Women tend to have relatively more adipose tissue in the hips and thighs compared to men who tend to have abdominal adiposity^33^.

The changing relationship of BMI and adiposity (fat mass index or percentage body fat) with age among adults has been suggested in several studies^1,6,7,30,34,35^. Previous results showing that the correlation between BMI and percentage body fat is weaker with increasing age are in agreement with our findings^1^. BMI was a suboptimal marker for percentage body fat in older adults, particulary in those with BMI ≥ 30 kg/m^2^^35^. Meanwhile, a study based on KNHANES did not find weakening correlation between BMI and percentage body fat with age^30^ and a study in Switzerland found stronger correlation of fat mass with BMI in adults age 50–84 years than those aged 15–49 years^34^. These inconsistent findings may be caused by ethnic differences^30^, less detailed age categorization^30,34^, and relatively small number of study population (n = 226)^34^. Other studies that used a regression model to estimate the influence of age on the correlation between BMI and percentage body fat reported a 0.7–1.4% increase in percentage body fat per decade of age^6,7^. The underlying mechanisms are related to reduced physical activity, low protein intake, and altered sex hormone and cytokine metabolism resulting in gain in fat mass and loss of lean mass^36^. In addition, low-grade chronic dehydration and plasma hyperosmolar stress in the elderly may reduce the muscle strength and muscle mass with age^37^. Age-related intracellular dehydration can be caused by several reasons including reduced thirst sensation and decrease in ability to concentrate urine.

In this study, the correlation between BMI with fat mass index and percentage body fat was lower in Korean compared to other race-ethnicities. Contributing factors may be the relatively higher percentage body fat with low BMI in Asian^38^, and the narrower range of BMI in Korean population, as the correlation coefficient is influenced by the range of the exposure (Supplementary Fig. 9 and Supplementary Fig. 10). The changing relationship between BMI and fat mass index or percentage body fat with age showed different pattern by race-ethnicities. The weakest correlation between BMI and percentage body fat in women aged 50–59 years was observed in Black, Mexican–American and Korean, but not in White people. This pattern may be related to perimenopausal changes in body composition which usually leads to decrease in the lean mass and increase in the total fat mass^39^. Changes in body composition related to menopause could be different by race-ethnicities. In Women’s Health Across the Nation cohort, Japanese and Chinese women in menopause transition did not lose lean mass, unlike White and Black women^40^. In our study, lean mass did not decrease among Korean women even in the pre- and postmenopausal period, unlike women in other ethnicities who started to lose lean mass at ages 50–59 years. Moreover, Korean men aged 50–59 years old also had the weakest correlation between BMI and percentage body fat. Korean men showed a peak of BMI in 40–49 years, after which time it decreased, whereas men of other race/ethnic showed a peak in 60–69 years, which then decreased. The transitional period of men aged 50–59 years may increase heterogeneity of fat mass index and percentage body fat at a given BMI, lowering the degree of correlation. The different pattern of correlation by race/ethnicity may be attributable to different metabolism and basal concentration of testosterone by age, considering the anabolic effect of testosterone^41,42^.

We also investigated the correlation of BMI, fat mass index, percentage body fat, and WC/truncal fat mass with obesity-related biomarkers. Previous studies examined the relationship between BMI and obesity-related biomarkers such as lipids, CRP, glucose level, and insulin^43^. Among these biomarkers, insulin resistance by homeostasis model assessment (HOMA-IR) was most strongly correlated with BMI and percentage body fat with adjustment for age and sex^43^. In addition, BMI was superior over percentage body fat in relation to obesity-related biomarkers^43^, which is consistent with our results. A study for Taiwanese people aged 64 on average reported that BMI and WC predicted insulin resistance better than percentage body fat did^44^. However, the changing relationship with age and sex was not examined^43,44^. In our study, the correlation of BMI with biomarkers was comparable to that of WC/truncal fat mass and the relationship between BMI and biomarkers generally became weaker as age increases in both men and women. However, the correlation between BMI and insulin in Korean men seems similar throughout all age groups, which might be related to less fluctuation in absolute fat mass index (ranged 5.0–5.5 kg/m^2^ in Korean men vs. 7.0–9.1 kg/m^2^ in other race/ethnicities) by age.

The participants with chronic diseases had a higher BMI, fat mass index, percentage body fat and lean mass index (except for cancer patients) than those without. The correlation between BMI and fat mass index and percentage body fat did not vary appreciably by comorbidity status. Although it is uncertain that body composition accompanied by each comorbidity is a cause or consequence related to chronic conditions, obesity is a well-known risk factor for metabolic diseases, cancer and cardiovascular diseases^45^.

Our study has several limitations to be considered. First, there might be a measurement bias to compare the data from two different surveys, NHANES and KNHANES, even though same DXA instrument (QDR 4500A) was used to assess body composition. Thus, direct comparison between two data should be made cautiously, though the patterns within each dataset should be valid. Furthermore, previous studies reported that body composition measured by this DXA instrument (QDR 4500A) overestimated fat-free mass regardless of age, sex and ethnicity: 5% higher fat-free mass compared with measurements by criteria methods^46^. This systematic bias may affect the magnitude of correlations. Second, those with missing DXA variables are more likely to be older and obese, and thus the included population in our study was still less obese. Thus, NHANES conducted multiple imputation methods to overcome selection bias. Third, data from KNHANES could not represent other Asian sub-ethnic groups since body composition and its relationship with cardiometabolic risk within Asian groups are heterogenous^47^. Fourth, even though we categorized race/ethnicity into White, Black, Mexican–American, and Korean (Asian), there is substantial heterogeneity within the same race/ethnicity. Discrete race/ethnic groups could not fully reflect variation in race/ethnicity. Fifth, we did not consider various confounding factors which may affect body weight and body composition such as smoking status and physical activity because our study focused on relationship between BMI and DXA-derived adiposity by age, sex, and race-ethnicities. Further research considering of these factors would be worthy.

## Conclusions

BMI is a good indicator for adiposity assessed by DXA, particularly in younger age groups, women, and Whites and Blacks. BMI has somewhat weaker correlations with fat mass index and percentage body fat in Korean populations, which may be driven by their lower body weights in general. BMI predicts adiposity-related biomarkers as well as fat mass index and slightly better than percentage body fat. The correlations between BMI with biomarkers generally goes down with increasing age, but this pattern is seen with fat mass index and percentage body fat as well. Thus, these more technically advanced measures will not necessarily be superior to BMI in most circumstances, particularly in epidemiological studies. When BMI, as an indicator for body fatness is used, the different context depending on age, sex, and race/ethnicities should be considered.

## Supplementary Information

Supplementary Information.

## Data Availability

The datasets generated and/or analysed during the current study are available in the Centers for Disease Control and Prevention for NHANES [https://www.cdc.gov/nchs/nhanes/index.htm] and Korea Disease Control and Prevention Agency for KNHANES [https://knhanes.kdca.go.kr/knhanes/eng/index.do].

## Abbreviations

BMI: Body mass index
DXA: Dual-energy X-ray absorptiometry
NHANES: National Health and Nutrition Examination Survey
FMI: Fat mass index
PBF: Percentage body fat
TC: Total cholesterol
LDL-C: Low-density lipoprotein cholesterol
HDL-C: High-density lipoprotein cholesterol
CRP: C-reactive protein
SE: Standard error

## References

[1] Flegal KM (2009). Comparisons of percentage body fat, body mass index, waist circumference, and waist-stature ratio in adults. Am. J. Clin. Nutr..

[2] Grier T, Canham-Chervak M, Sharp M, Jones BH (2015). Does body mass index misclassify physically active young men. Prev. Med. Rep..

[3] Akindele MO, Phillips JS, Igumbor EU (2016). The relationship between body fat percentage and body mass index in overweight and obese individuals in an urban African setting. J. Public Health Africa.

[4] Ranasinghe C (2013). Relationship between Body Mass Index (BMI) and body fat percentage, estimated by bioelectrical impedance, in a group of Sri Lankan adults: A cross sectional study. BMC Public Health.

[5] Misra P, Singh AK, Archana S, Lohiya A, Kant S (2019). Relationship between body mass index and percentage of body fat, estimated by bio-electrical impedance among adult females in a rural community of North India: A cross-sectional study. J. Postgrad. Med..

[6] Meeuwsen S, Horgan GW, Elia M (2010). The relationship between BMI and percent body fat, measured by bioelectrical impedance, in a large adult sample is curvilinear and influenced by age and sex. Clin. Nutr..

[7] Gallagher D (1996). How useful is body mass index for comparison of body fatness across age, sex, and ethnic groups?. Am. J. Epidemiol..

[8] Wang J (1994). Asians have lower body mass index (BMI) but higher percent body fat than do whites: Comparisons of anthropometric measurements. Am. J. Clin. Nutr..

[9] Wagner DR, Heyward VH (2000). Measures of body composition in blacks and whites: A comparative review. Am. J. Clin. Nutr..

[10] Wu CH (2007). Truncal fat in relation to total body fat: Influences of age, sex, ethnicity and fatness. Int. J. Obes..

[11] Misra A, Wasir JS, Vikram NK (2005). Waist circumference criteria for the diagnosis of abdominal obesity are not applicable uniformly to all populations and ethnic groups. Nutrition.

[12] Rickenbacher M (2022). The role of body height as a co-factor of excess weight in Switzerland. Am. J. Hum. Biol..

[13] Group, D. P. C. (2005). Weight-height relationships and body mass index: Some observations from the diverse populations collaboration. Am. J. Phys. Anthropol..

[14] Ashwell M, Gibson S (2014). A proposal for a primary screening tool: 'Keep your waist circumference to less than half your height'. BMC Med..

[15] Al-Goblan AS, Al-Alfi MA, Khan MZ (2014). Mechanism linking diabetes mellitus and obesity. Diabetes Metab. Syndr. Obes..

[16] Meigs JB (2006). Body mass index, metabolic syndrome, and risk of type 2 diabetes or cardiovascular disease. J. Clin. Endocrinol. Metab..

[17] Romero-Corral A (2009). Normal weight obesity: A risk factor for cardiometabolic dysregulation and cardiovascular mortality. Eur. Heart J..

[18] De Lorenzo A (2013). Adiposity rather than BMI determines metabolic risk. Int. J. Cardiol..

[19] Lee DH (2018). Predicted lean body mass, fat mass, and all cause and cause specific mortality in men: Prospective US cohort study. BMJ.

[20] Hainer V, Aldhoon-Hainerová I (2013). Obesity paradox does exist. Diabetes Care.

[21] Paulose-Ram R, Burt V, Broitman L, Ahluwalia N (2017). Overview of Asian American data collection, release, and analysis: National Health and Nutrition Examination Survey 2011–2018. Am. J. Public Health.

[22] Hong S (2011). Characteristics of body fat, body fat percentage and other body composition for Koreans from KNHANES IV. J. Korean Med. Sci..

[23] Lindsay M. Monte and Hyon B. Shin. 2022. 20.6 Million People in the U.S. Identify as Asian, Native Hawaiian or Pacific Islander. Census Bureau. May 25 https://www.census.gov/library/stories/2022/05/aanhpi-population-diverse-geographically-dispersed.html (2023).

[24] Weir, C. B. & Jan, A. BMI classification percentile and cut off points. [Updated 2020 Jul 10]. In *StatPearls*. https://www.ncbi.nlm.nih.gov/books/NBK541070/ (StatPearls Publishing, 2020). 31082114

[25] Kelly TL, Wilson KE, Heymsfield SB (2009). Dual energy X-Ray absorptiometry body composition reference values from NHANES. PLoS ONE.

[26] Weber DR, Leonard MB, Shults J, Zemel BS (2014). A comparison of fat and lean body mass index to BMI for the identification of metabolic syndrome in children and adolescents. J. Clin. Endocrinol. Metab..

[27] National Health and Nutrition Examination Survey: The 1999–2006 Dual Energy X-ray Absorptiometry (DXA) Multiple Imputation Data Files and Technical Documentation https://wwwn.cdc.gov/Nchs/Nhanes/Dxa/Dxa.aspx.

[28] Schenker N (2011). Multiple imputation of missing dual-energy X-ray absorptiometry data in the National Health and Nutrition Examination Survey. Stat. Med..

[29] Lindsay RS (2001). Body mass index as a measure of adiposity in children and adolescents: Relationship to adiposity by dual energy x-ray absorptiometry and to cardiovascular risk factors. J. Clin. Endocrinol. Metab..

[30] Kim SG (2015). Relationship between indices of obesity obtained by anthropometry and dual-energy X-ray absorptiometry: The Fourth and Fifth Korea National Health and Nutrition Examination Survey (KNHANES IV and V, 2008–2011). Obes. Res. Clin. Pract..

[31] Steinberger J (2005). Comparison of body fatness measurements by BMI and skinfolds vs dual energy X-ray absorptiometry and their relation to cardiovascular risk factors in adolescents. Int. J. Obes..

[32] Jackson AS (2002). The effect of sex, age and race on estimating percentage body fat from body mass index: The Heritage Family Study. Int. J. Obes..

[33] Karastergiou K, Smith SR, Greenberg AS, Fried SK (2012). Sex differences in human adipose tissues—The biology of pear shape. Biol. Sex Differ..

[34] Morabia A, Ross A, Curtin F, Pichard C, Slosman DO (1999). Relation of BMI to a dual-energy X-ray absorptiometry measure of fatness. Br. J. Nutr..

[35] Batsis JA (2016). Diagnostic accuracy of body mass index to identify obesity in older adults: NHANES 1999–2004. Int. J. Obes..

[36] Batsis JA, Villareal DT (2018). Sarcopenic obesity in older adults: Aetiology, epidemiology and treatment strategies. Nat. Rev. Endocrinol..

[37] Lorenzo I, Serra-Prat M, Yébenes JC (2019). The role of water homeostasis in muscle function and frailty: A review. Nutrients.

[38] Carpenter CL (2013). Body fat and body-mass index among a multiethnic sample of college-age men and women. J. Obes..

[39] Heymsfield SB (1994). Menopausal changes in body composition and energy expenditure. Exp. Gerontol..

[40] Greendale GA (2019). Changes in body composition and weight during the menopause transition. JCI Insight.

[41] Eendebak RJAH (2017). Ethnic differences in male reproductive hormones and relationships with adiposity and insulin resistance in older men. Clin. Endocrinol..

[42] Jakobsson J (2006). Large differences in testosterone excretion in Korean and Swedish men are strongly associated with a UDP-glucuronosyl transferase 2B17 polymorphism. J. Clin. Endocrinol. Metab..

[43] Bosy-Westphal A (2006). Value of body fat mass vs anthropometric obesity indices in the assessment of metabolic risk factors. Int. J. Obes..

[44] Cheng Y-H (2017). Body mass index and waist circumference are better predictors of insulin resistance than total body fat percentage in middle-aged and elderly Taiwanese. Medicine.

[45] Guh DP (2009). The incidence of co-morbidities related to obesity and overweight: A systematic review and meta-analysis. BMC Public Health.

[46] Schoeller DA (2005). QDR 4500A dual-energy X-ray absorptiometer underestimates fat mass in comparison with criterion methods in adults. Am. J. Clin. Nutr..

[47] Nazare JA (2012). Ethnic influences on the relations between abdominal subcutaneous and visceral adiposity, liver fat, and cardiometabolic risk profile: The international study of prediction of intra-abdominal adiposity and its relationship with cardiometabolic risk/intra-abdominal adiposity. Am. J. Clin. Nutr..

