# Supplementary material for: Different correlation of body mass index with body fatness and obesity-related biomarker according to age, sex and race-ethnicity
Source: Sci Rep. 2023 Mar 1;13:3472. doi: 10.1038/s41598-023-30527-w (PMC9977890; doi:10.1038/s41598-023-30527-w)

**Supplementary Table 1. Baseline characteristics of study participants, National Health and Nutrition Examination Survey (US, 1999-2006) and Korea National Health and Nutrition Examination Survey (Korea, 2008-2011)**

|  | **NHANES** | | | **Korea NHANES** | | |
| --- | --- | --- | --- | --- | --- | --- |
|  | **Men** | **Women** |  | **Men** | **Women** |  |
|  | **Mean (SE) or W%** | | ***p*-value** | **Mean (SE) or W%** | | ***p*-value** |
| Unweighted N | 9,141 | 8,920 |  | 8,107 | 10,754 |  |
| Sex | 49.3% | 50.7% |  | 49.7% | 50.3% |  |
| Age, years | 43.2 (0.3) | 45.3 (0.3) | < 0.001 | 43.2 (0.3) | 45.5 (0.3) | < 0.001 |
| 18-29 | 23.6% | 20.1% | < 0.001 | 22.4% | 19.7% | < 0.001 |
| 30-39 | 20.7% | 19.4% |  | 22.1% | 20.2% |  |
| 40-49 | 21.9% | 22.3% |  | 22.2% | 21.6% |  |
| 50-59 | 16.7% | 17.2% |  | 17.1% | 17.0% |  |
| 60-69 | 10.3% | 11.3% |  | 9.7% | 10.7% |  |
| ≥70 | 6.8% | 9.6% |  | 6.5% | 10.7% |  |
| BMI*, kg/m^2^ | 27.9 (0.1) | 28.2 (0.1) | 0.014 | 24.0 (0.1) | 23.1 (0.1) | < 0.001 |
| < 18.5 | 1.4% | 2.7% | < 0.001 | 3.4% | 6.8% | < 0.001 |
| 18.5-24.9 | 29.9% | 36.6% |  | 35.8% | 45.8% |  |
| 25-29.9 | 39.9% | 27.6% |  | 25.1% | 20.8% |  |
| ≥ 30 | 28.8% | 33.2% |  | 35.7% | 26.6% |  |
| WC*, cm | 99.3 (0.3) | 93.0 (0.3) | < 0.001 | 83.7 (0.2) | 77.6 (0.2) | < 0.001 |
| ≥ 102 cm (men)  or ≥ 88 cm (women) | 39.5% | 57.8% | < 0.001 | 24.1% | 39.0% | < 0.001 |
| Height, cm | 176.3 (0.1) | 162.3 (0.1) | < 0.001 | 170.7 (0.1) | 157.2 (0.1) | < 0.001 |
| Waist-height ratio | 0.56 | 0.57 | < 0.001 | 0.49 | 0.50 | < 0.001 |
| Fat mass, kg | 25.2 (0.2) | 30.7 (0.2) | < 0.001 | 15.5 (0.1) | 18.9 (0.1) | < 0.001 |
| FMI, kg/m2 | 8.1 (0.06) | 11.6 (0.09) | < 0.001 | 5.3 (0.03) | 7.7 (0.4) | < 0.001 |
| PBF, % | 27.7 (0.1) | 39.6 (0.1) | < 0.001 | 22.0 (0.1) | 32.9 (0.1) | < 0.001 |
| Lean body mass, kg | 59.9 (0.2) | 42.1 (0.1) | < 0.001 | 51.1 (0.1) | 35.8 (0.07) | < 0.001 |
| LMI, kg/m2 | 19.2 (0.05) | 15.9 (0.05) | < 0.001 | 17.5 (0.03) | 14.5 (0.03) | < 0.001 |
| Truncal fat, kg | 13.1 (0.08) | 14.5 (0.09) | < 0.001 | 8.4 (0.06) | 9.4 (0.06) | < 0.001 |
| ASMI, kg/m2 | 8.6 (0.02) | 6.7 (0.02) | < 0.001 | 7.7 (0.02) | 5.9 (0.01) | < 0.001 |
| Ethnicity |  |  |  |  |  |  |
| Non-Hispanic white | 71.5% | 70.7% | < 0.001 | NA | NA | NA |
| Non-Hispanic black | 10.3% | 11.7% |  | NA | NA | NA |
| Mexican-American | 8.4% | 6.8% |  | NA | NA | NA |
| Other Hispanic | 4.9% | 5.6% |  | NA | NA | NA |
| Other | 4.9% | 5.1% |  | NA | NA | NA |
| Smoking status^a^ |  |  |  | NA | NA | NA |
| Never | 43.2% | 57.0% | < 0.001 | 40.2% | 90.5% | < 0.001 |
| Past | 28.7% | 22.2% |  | 13.7% | 2.9% |  |
| Current | 28.1% | 20.8% |  | 46.1% | 6.6% |  |
| MVPA, yes | 68.5% | 65.3% | < 0.001 | 26.8% | 21.8% | < 0.001 |
| Blood pressure^a^ |  |  |  |  |  |  |
| SBP, mmHg | 123.0 (0.2) | 120.9 (0.2) | < 0.001 | 120.4 (0.3) | 115.5 (0.3) | < 0.001 |
| DBP, mmHg | 73.1 (0.1) | 70.7 (0.1) | < 0.001 | 79.9 (0.2) | 74.0 (0.2) | < 0.001 |
| Obesity-related biomarkers |  |  |  |  |  |  |
| Total cholesterol, mg/dL | 199.4 (0.5) | 201.5 (0.4) | 0.001 | 185.6 (0.6) | 185.8 (0.5) | < 0.001 |
| LDL-C, mg/dL | 155.6 (2.2) | 129.9 (1.9) | 0.005 | 110.3 (0.5) | 113.1 (0.4) | < 0.001 |
| HDL-C, mg/dL | 47.1 (0.1) | 57.6 (0.2) | < 0.001 | 45.8 (0.2) | 50.9 (0.2) | < 0.001 |
| TG, mg/dL^b^ | 128.0 | 109.8 | < 0.001 | 126.2 | 93.0 | < 0.001 |
| CRP, mg/dL | 0.315 (0.008) | 0.577 (0.01) | < 0.001 | NA | NA | NA |
| FBG, mg/dL | 103.3 (0.5) | 99.6 (0.5) | < 0.001 | 98.0 (0.3) | 94.8 (0.3) | < 0.001 |
| HbA1c, %^c^ | 5.5 (0.01) | 5.4 (0.01) | 0.002 | 6.7 (0.07) | 6.6 (0.07) | 0.002 |
| Insulin, μU/ml^c^ | 12.5 (0.2) | 11.4 (0.2) | < 0.001 | 10.1 (0.1) | 10.1 (0.1) | 0.325 |
| Chronic diseases |  |  |  |  |  |  |
| No | 79.5% | 69.3% | < 0.001 | 89.1% | 84.7% | < 0.001 |
| Yes^d^ | 20.5% | 30.7% |  | 10.9% | 15.3% |  |
| Cancer | 5.9% | 8.8% | < 0.001 | 1.6% | 3.2% | < 0.001 |
| Coronary heart disease | 6.4% | 4.6% | < 0.001 | 1.7% | 1.7% | 0.322 |
| Congestive heart failure | 2.2% | 1.7% | < 0.001 | NA | NA | NA |
| Stroke | 1.7% | 2.5% | < 0.001 | 1.5% | 1.1% | 0.026 |
| Thyroid disease | 3.0% | 13.1% | < 0.001 | 0.9% | 5.4% | < 0.001 |
| Chronic pulmonary disease | 5.2% | 9.3% | < 0.001 | 4.0% | 4.4% | 0.233 |
| Liver disease | 3.7% | 2.6% | < 0.001 | 2.3% | 1.5% | < 0.001 |

WC, waist circumference; FMI, fat mass index; PBF, percentage body fat; LMI, lean mass index; MVPA, Moderate to vigorous physical activity; SBP, systolic blood pressure; DBP, diastolic blood pressure; LDL-C, low density lipoprotein cholesterol; HDL-C, high density lipoprotein cholesterol; TG, triglyceride; CRP, C-reactive protein; FBG, fasting blood glucose; NA, not applicable

^a^ Information on smoking status and blood pressure measurement was available in 16,020 and 16,962 participants, respectively

^b^ Geometric mean

^c^ Information on HbA1c and insulin was available in 3748 and 15823 Korean participants, respectively.

^d^ The presence of chronic diseases was defined when individuals have as least one disease among cancer, coronary heart disease, congestive heart failure, stroke, thyroid disease, chronic pulmonary disease, and liver disease.

*For Koreans, BMI was categorized into < 18.5, 18.5-22.9, 23-24.9, and ≥25 kg/m^2^ and cut-off of WC was 90cm in men and 80cm in women.

**Supplementary Table 2. Baseline characteristics of participants included versus excluded.**

|  | **NHANES** | | | **Korea NHANES** | | |
| --- | --- | --- | --- | --- | --- | --- |
|  | **Inclusion**  **(n=18,061)** | **Exclusion**  **(n=2,317)** |  | **Inclusion**  **(n=18,861)** | **Exclusion**  **(n=640)** |  |
|  | **Mean (SE) or W%** | | ***p*-value** | **Mean (SE) or W%** | | ***p*-value** |
| Sex, male | 49.4% | 32.5% | < 0.001 | 49.7% | 48.3% | 0.803 |
| Age, years | 43.9 (0.3) | 43.1 (0.8) | < 0.001 | 44.4 (0.3) | 46.1 (2.1) | 0.208 |
| 18-29 | 22.5% | 32.9% | < 0.001 | 21.0% | 13.3% | 0.074 |
| 30-39 | 20.6% | 22.0% |  | 22.2% | 27.3% |  |
| 40-49 | 21.9% | 12.8% |  | 21.9% | 26.6% |  |
| 50-59 | 16.6% | 9.7% |  | 17.1% | 8.9% |  |
| 60-69 | 10.8% | 6.9% |  | 10.2% | 10.3% |  |
| ≥70 | 7.6% | 15.7% |  | 8.6% | 13.6% |  |
| BMI*, kg/m^2^ | 28.0 (0.1) | 29.6 (0.3) | < 0.001 | 23.6 (0.03) | 23.8 (0.6) | 0.908 |
| < 18.5 | 2.1% | 2.2% | 0.001 | 5.1% | 4.4% | 0.966 |
| 18.5-24.9 | 33.4% | 29.0% |  | 63.8% | 61.8% |  |
| 25-29.9 | 33.5% | 29.7% |  | 27.2% | 29.9% |  |
| ≥ 30 | 31.0% | 39.1% |  | 4.0% | 4.0% |  |
| WC*, cm | 96.0 (0.3) | 99.9 (0.7) | < 0.001 | 80.7 (0.1) | 92.6 (3.5) | 0.003 |
| Height, cm | 169.3 (0.1) | 165.9 (0.4) | < 0.001 | 170.7 (0.1) | 157.2 (0.1) | < 0.001 |
| WHtR | 0.57 | 0.61 |  | 0.49 | 0.53 |  |
| Fat mass, kg | 27.9 (0.2) | 31.0 (0.8) | < 0.001 | 17.3 (0.1) | 17.7 (0.7) | 0.645 |
| FMI, kg/m2 | 9.9 (0.1) | 11.3 (0.3) | < 0.001 | 6.5 (0.03) | 6.8 (0.3) | 0.573 |
| PBF, % | 33.7 (0.1) | 35.8 (0.5) | < 0.001 | 27.5 (0.1) | 28.3 (0.9) | 0.590 |
| Lean body mass, kg | 53.4 (0.1) | 53.2 (0.9) | 0.224 | 43.4 (0.1) | 43.2 (1.5) | 0.776 |
| LMI, kg/m2 | 18.4 (0.04) | 19.0 (0.3) | < 0.001 | 16.0 (0.03) | 15.9 (0.4) | 0.769 |
| Truncal fat, kg | 13.8 (0.1) | 15.5 (0.4) | < 0.001 | 9.0 (0.1) | 9.3 (0.5) | 0.468 |
| ASMI, kg/m2 | 8.1 (0.02) | 8.3 (0.1) | 0.109 | 6.8 (0.02) | 6.7 (0.2) | 0.826 |
| Ethnicity |  |  |  |  |  |  |
| Non-Hispanic white | 71.1% | 62.5% | <0.001 | NA | NA | NA |
| Non-Hispanic black | 11.0% | 15.8% |  | NA | NA | NA |
| Mexican-American | 7.0% | 9.5% |  | NA | NA | NA |
| Other Hispanic | 5.3% | 5.7% |  | NA | NA | NA |
| Other | 5.1% | 6.5% |  | NA | NA | NA |

**Supplementary Table 3. Pearson correlation of waist circumference, percentage body fat, lean body mass index and fat mass index with waist-height ratio by sex and age group in National Health and Nutrition Examination Survey (NHANES) and Korea NHANES**

| **Age group (years)** | **WC** | **FMI** | **PBF** | **TF** | **LMI** | **ASMI** |
| --- | --- | --- | --- | --- | --- | --- |
| **NHANES** |  |  |  |  |  |  |
| Men | 0.958 | 0.941 | 0.859 | 0.915 | 0.758 | 0.606 |
| 18-29 | 0.966 | 0.961 | 0.888 | 0.942 | 0.818 | 0.727 |
| 30-39 | 0.954 | 0.950 | 0.849 | 0.921 | 0.801 | 0.695 |
| 40-49 | 0.957 | 0.944 | 0.834 | 0.914 | 0.833 | 0.720 |
| 50-59 | 0.940 | 0.935 | 0.818 | 0.881 | 0.807 | 0.678 |
| 60-69 | 0.947 | 0.928 | 0.799 | 0.880 | 0.796 | 0.666 |
| ≥70 | 0.934 | 0.903 | 0.781 | 0.843 | 0.745 | 0.615 |
| Women | 0.970 | 0.904 | 0.798 | 0.904 | 0.817 | 0.727 |
| 18-29 | 0.972 | 0.923 | 0.821 | 0.923 | 0.859 | 0.795 |
| 30-39 | 0.971 | 0.922 | 0.810 | 0.920 | 0.855 | 0.784 |
| 40-49 | 0.971 | 0.915 | 0.793 | 0.924 | 0.864 | 0.804 |
| 50-59 | 0.969 | 0.899 | 0.768 | 0.905 | 0.853 | 0.786 |
| 60-69 | 0.964 | 0.874 | 0.726 | 0.877 | 0.836 | 0.759 |
| ≥70 | 0.955 | 0.841 | 0.683 | 0.826 | 0.783 | 0.689 |
| **Korea NHANES** |  |  |  |  |  |  |
| Men | 0.939 | 0.783 | 0.664 | 0.771 | 0.631 | 0.479 |
| 18-29 | 0.963 | 0.871 | 0.748 | 0.868 | 0.721 | 0.649 |
| 30-39 | 0.948 | 0.831 | 0.692 | 0.808 | 0.698 | 0.609 |
| 40-49 | 0.945 | 0.812 | 0.664 | 0.801 | 0.678 | 0.579 |
| 50-59 | 0.931 | 0.752 | 0.588 | 0.722 | 0.630 | 0.531 |
| 60-69 | 0.944 | 0.763 | 0.635 | 0.734 | 0.651 | 0.530 |
| ≥70 | 0.952 | 0.819 | 0.699 | 0.791 | 0.668 | 0.526 |
| Women | 0.954 | 0.787 | 0.616 | 0.781 | 0.698 | 0.560 |
| 18-29 | 0.960 | 0.837 | 0.669 | 0.838 | 0.716 | 0.653 |
| 30-39 | 0.961 | 0.830 | 0.632 | 0.831 | 0.753 | 0.690 |
| 40-49 | 0.957 | 0.800 | 0.601 | 0.804 | 0.697 | 0.611 |
| 50-59 | 0.951 | 0.773 | 0.553 | 0.743 | 0.633 | 0.527 |
| 60-69 | 0.946 | 0.768 | 0.581 | 0.737 | 0.623 | 0.485 |
| ≥70 | 0.946 | 0.820 | 0.698 | 0.768 | 0.610 | 0.437 |

**Supplementary Table 4.** Partial correlation of waist circumference, percentage body fat, lean body mass index and fat mass index with body mass index by presence of comorbidities adjusted for age (as continuous variable)

|  | **WC, cm** | **BMI, kg/m^2^** | **FMI, kg/m2** | **PBF, %** | **LMI, kg/m^2^** | **WC** | **FMI** | **PBF** | **LMI** |
| --- | --- | --- | --- | --- | --- | --- | --- | --- | --- |
| **Presence of comorbidities** | **Mean (SE)** | **Mean (SE)** | **Mean (SE)** | **Mean (SE)** | **Mean (SE)** | **Correlation coefficients with BMI** | | | |
| Comorbidities |  |  |  |  |  |  |  |  |  |
| Men |  |  |  |  |  |  |  |  |  |
| No | 98.0 (0.3) | 27.7 (0.1) | 7.9 (0.1) | 27.2 (0.1) | 19.2 (0.1) | 0.936 | 0.947 | 0.925 | 0.927 |
| Yes | 104.3 (0.5) | 28.8 (0.2) | 8.9 (0.1) | 30.0 (0.2) | 19.3 (0.1) | 0.933 | 0.949 | 0.927 | 0.930 |
| Women |  |  |  |  |  |  |  |  |  |
| No | 91.4 (0.4) | 27.8 (0.1) | 11.3 (0.1) | 39.0 (0.2) | 15.9 (0.1) | 0.919 | 0.977 | 0.956 | 0.930 |
| Yes | 96.5 (0.4) | 29.1 (0.2) | 12.4 (0.1) | 41.1 (0.2) | 16.1 (0.1) | 0.908 | 0.979 | 0.955 | 0.932 |
| Cancer |  |  |  |  |  |  |  |  |  |
| Men |  |  |  |  |  |  |  |  |  |
| No | 99.0 (0.3) | 27.9 (0.1) | 8.1 (0.1) | 27.6 (0.1) | 19.3 (0.1) | 0.936 | 0.948 | 0.926 | 0.929 |
| Yes | 103.7 (0.6) | 28.0 (0.2) | 8.7 (0.1) | 30.2 (0.2) | 18.6 (0.1) | 0.934 | 0.936 | 0.919 | 0.895 |
| Women |  |  |  |  |  |  |  |  |  |
| No | 92.8 (0.4) | 28.2 (0.1) | 11.6 (0.1) | 39.5 (0.2) | 16.0 (0.1) | 0.918 | 0.978 | 0.957 | 0.932 |
| Yes | 95.1 (0.7) | 28.1 (0.3) | 11.8 (0.2) | 40.6 (0.3) | 15.7 (0.1) | 0.895 | 0.977 | 0.949 | 0.928 |
| Coronary heart disease |  |  |  |  |  |  |  |  |  |
| Men |  |  |  |  |  |  |  |  |  |
| No | 98.8 (0.3) | 27.9 (0.1) | 8.0 (01) | 27.5 (0.1) | 19.2 (0.1) | 0.936 | 0.947 | 0.926 | 0.928 |
| Yes | 105.5 (0.7) | 29.1 (0.3) | 9.2 (0.2) | 30.7 (0.3) | 19.3 (0.1) | 0.930 | 0.943 | 0.921 | 0.919 |
| Women |  |  |  |  |  |  |  |  |  |
| No | 92.7 (0.3) | 28.1 (0.1) | 11.6 (0.1) | 39.5 (0.1) | 16.0 (0.1) | 0.916 | 0.978 | 0.956 | 0.932 |
| Yes | 99.2 (0.8) | 29.7 (0.4) | 13.0 (0.2) | 42.5 (0.3) | 16.1 (0.2) | 0.894 | 0.971 | 0.949 | 0.915 |
| Congestive heart failure |  |  |  |  |  |  |  |  |  |
| Men |  |  |  |  |  |  |  |  |  |
| No | 99.1 (0.3) | 27.9 (0.1) | 8.1 (0.1) | 27.7 (0.1) | 19.2 (0.1) | 0.936 | 0.947 | 0.926 | 0.928 |
| Yes | 107.2 (1.4) | 29.5 (0.5) | 9.5 (0.3) | 31.1 (0.6) | 19.4 (0.2) | 0.938 | 0.941 | 0.914 | 0.919 |
| Women |  |  |  |  |  |  |  |  |  |
| No | 92.8 (0.3) | 28.1 (0.1) | 11.6 (0.1) | 39.6 (0.1) | 15.9 (0.1) | 0.916 | 0.978 | 0.956 | 0.931 |
| Yes | 104.0 (1.6) | 32.1 (0.9) | 14.5 (0.6) | 43.5 (0.6) | 17.1 (0.3) | 0.880 | 0.983 | 0.958 | 0.947 |
| Stroke |  |  |  |  |  |  |  |  |  |
| Men |  |  |  |  |  |  |  |  |  |
| No | 99.1 (0.3) | 27.9 (0.1) | 8.1 (0.1) | 27.2 (0.1) | 19.2 (0.1) | 0.936 | 0.947 | 0.926 | 0.928 |
| Yes | 106.3 (1.2) | 29.6 (0.5) | 9.7 (0.3) | 31.7 (0.4) | 19.2 (0.2) | 0.926 | 0.941 | 0.920 | 0.911 |
| Women |  |  |  |  |  |  |  |  |  |
| No | 92.8 (0.3) | 28.1 (0.1) | 11.6 (0.1) | 39.6 (0.1) | 16.0 (0.1) | 0.917 | 0.978 | 0.956 | 0.932 |
| Yes | 99.2 (1.0) | 29.5 (0.5) | 12.7 (0.3) | 41.6 (0.5) | 16.2 (0.2) | 0.880 | 0.970 | 0.951 | 0.912 |
| Thyroid disease |  |  |  |  |  |  |  |  |  |
| Men |  |  |  |  |  |  |  |  |  |
| No | 99.1 (0.3) | 27.9 (0.1) | 8.1 (0.1) | 27.7 (0.1) | 19.2 (0.1) | 0.937 | 0.947 | 0.926 | 0.927 |
| Yes | 104.2 (1.3) | 28.9 (0.5) | 8.9 (0.3) | 29.7 (0.6) | 19.4 (0.2) | 0.925 | 0.949 | 0.914 | 0.933 |
| Women |  |  |  |  |  |  |  |  |  |
| No | 92.4 (0.4) | 28.0 (0.1) | 11.5 (0.1) | 39.3 (0.2) | 15.9 (0.1) | 0.917 | 0.977 | 0.956 | 0.931 |
| Yes | 97.0 (0.5) | 29.4 (0.3) | 12.7 (0.2) | 41.5 (0.2) | 16.2 (0.1) | 0.907 | 0.980 | 0.954 | 0.936 |
| Chronic pulmonary disease |  |  |  |  |  |  |  |  |  |
| Men |  |  |  |  |  |  |  |  |  |
| No | 99.0 (0.3) | 27.9 (0.1) | 8.0 (0.1) | 27.6 (0.1) | 19.2 (0.1) | 0.936 | 0.947 | 0.925 | 0.926 |
| Yes | 104.9 (1.1) | 29.1 (0.4) | 9.1 (0.2) | 29.9 (0.4) | 19.4 (0.2) | 0.945 | 0.958 | 0.938 | 0.945 |
| Women |  |  |  |  |  |  |  |  |  |
| No | 92.5 (0.4) | 28.0 (0.1) | 11.5 (0.1) | 39.5 (0.1) | 15.9 (0.1) | 0.915 | 0.977 | 0.955 | 0.930 |
| Yes | 98.0 (0.7) | 29.8 (0.3) | 12.8 (0.2) | 41.2 (0.3) | 16.4 (0.1) | 0.915 | 0.981 | 0.959 | 0.939 |
| Liver disease |  |  |  |  |  |  |  |  |  |
| Men |  |  |  |  |  |  |  |  |  |
| No | 99.1 (0.3) | 27.9 (0.1) | 8.1 (0.1) | 27.7 (0.1) | 19.2 (0.1) | 0.937 | 0.947 | 0.926 | 0.927 |
| Yes | 102.7 (1.1) | 28.5 (0.4) | 8.6 (0.2) | 28.9 (0.4) | 19.4 (0.2) | 0.928 | 0.948 | 0.928 | 0.932 |
| Women |  |  |  |  |  |  |  |  |  |
| No | 92.9 (0.3) | 28.1 (0.1) | 11.6 (0.1) | 39.6 (0.1) | 16.0 (0.1) | 0.915 | 0.978 | 0.956 | 0.931 |
| Yes | 97.3 (1.5) | 29.5 (0.7) | 12.5 (0.4) | 40.5 (0.6) | 16.4 (0.3) | 0.936 | 0.979 | 0.961 | 0.929 |

WC, waist circumference; FMI, fat mass index; PBF, percentage body fat; LMI, lean mass index; BMI, body mass index

All *p* values < 0.001

**Supplementary Figure 1. Flow chart of the study population in National Health and Nutrition Examination Survey (NHANES)**


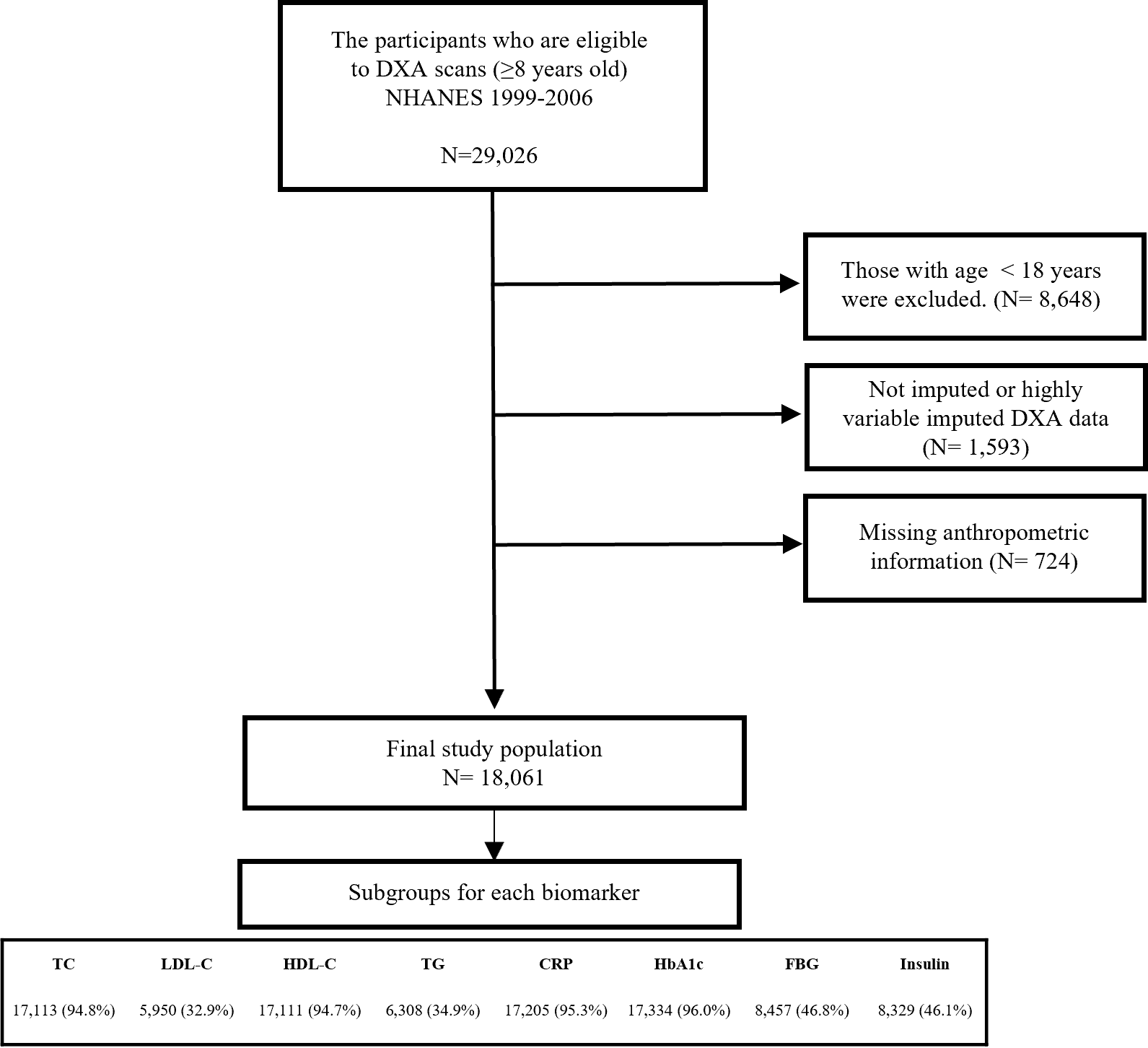


DXA, dual-energy X-ray absorptiometry TC, total cholesterol, LDL-C, low-density lipoprotein cholesterol, HDL-C, high-density lipoprotein cholesterol, TG, triglycerides, CRP, C-reactive protein, HbA1c, hemoglobinA1c, FBG, fasting blood glucose

**Supplementary Figure 2. Flow chart of the study population in Korea National Health and Nutrition Examination Survey (KNHANES)**


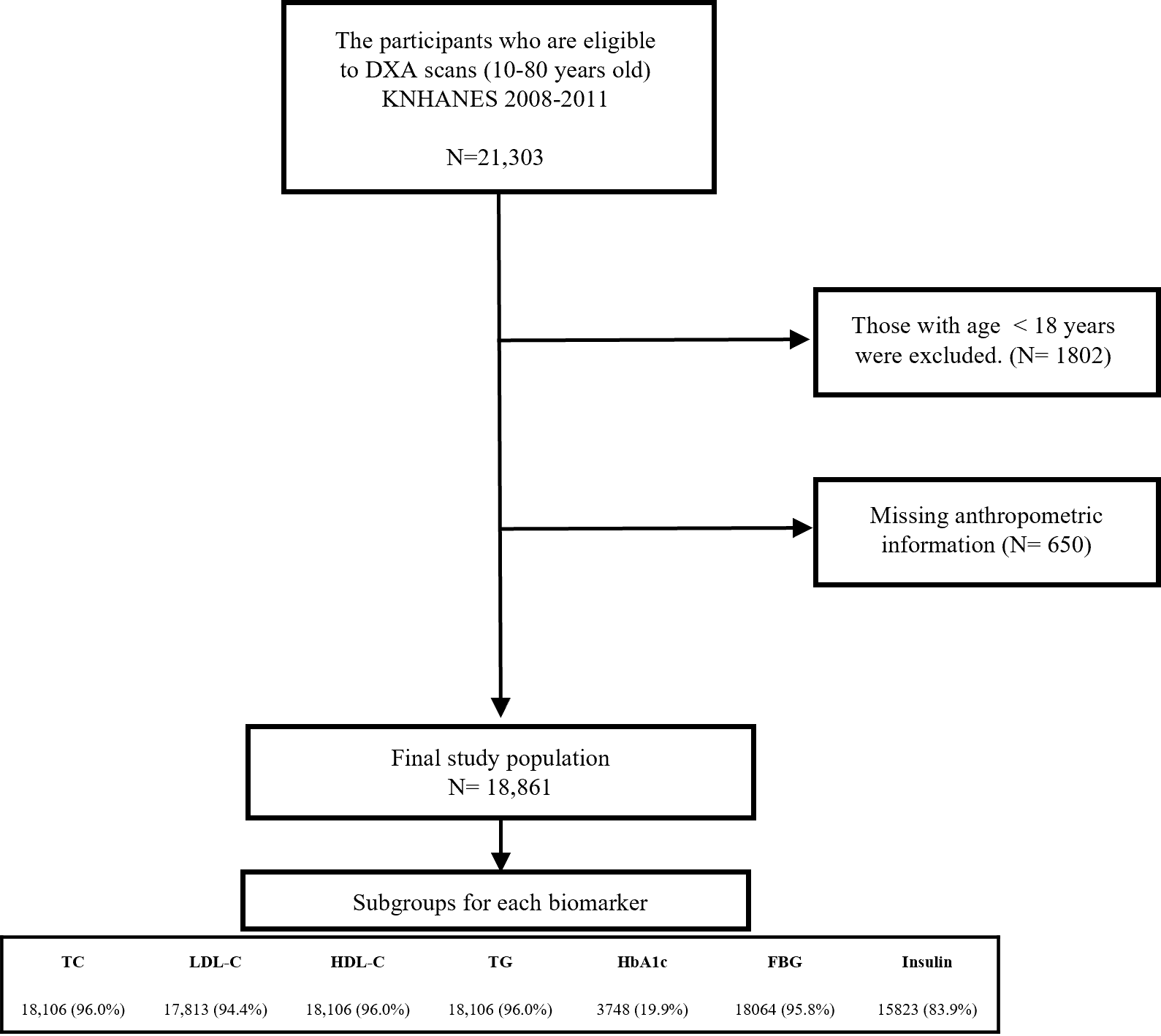


DXA, dual-energy X-ray absorptiometry TC, total cholesterol, LDL-C, low-density lipoprotein cholesterol, HDL-C, high-density lipoprotein cholesterol, TG, triglycerides HbA1c, hemoglobinA1c, FBG, fasting blood glucose

**Supplementary Figure 3. Scatter plots for body mass index and fat mass index by sex and age groups**

Legend: The plots of A - F show relationships between body mass index and fat mass index (FMI) in (A) 18-29 years (B) 30-39 years (C) 40-49 years, (D) 50-59 years (E) 60-69 years, and (F) ≥ 70 years.


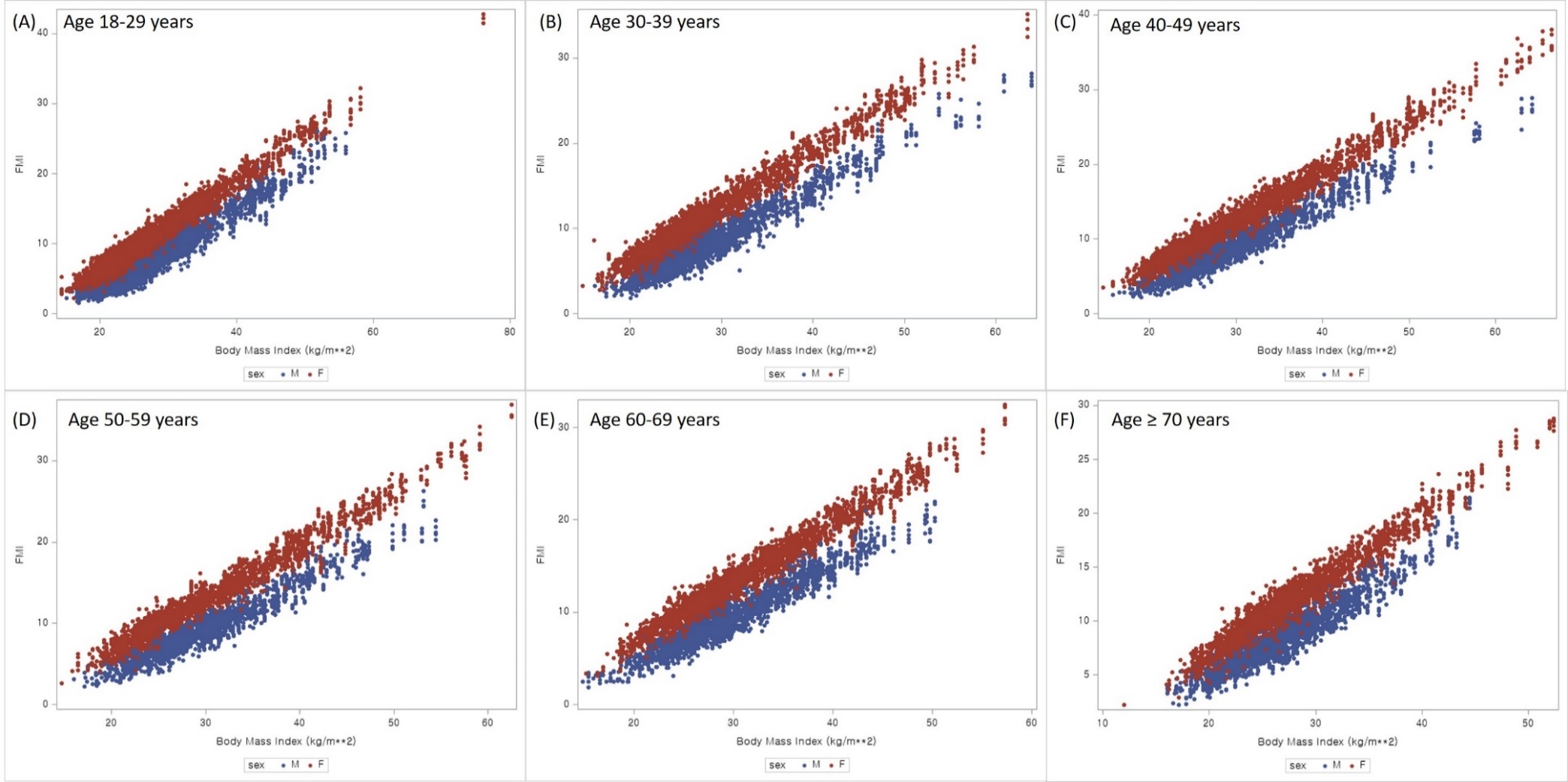


**Supplementary Figure 4. Scatter plots for body mass index and percentage body fat by sex and age groups**

Legend: The plots of A - F show relationships between body mass index and percentage body fat in (A) 18-29 years (B) 30-39 years (C) 40-49 years, (D) 50-59 years (E) 60-69 years, and (F) ≥ 70 years.

**
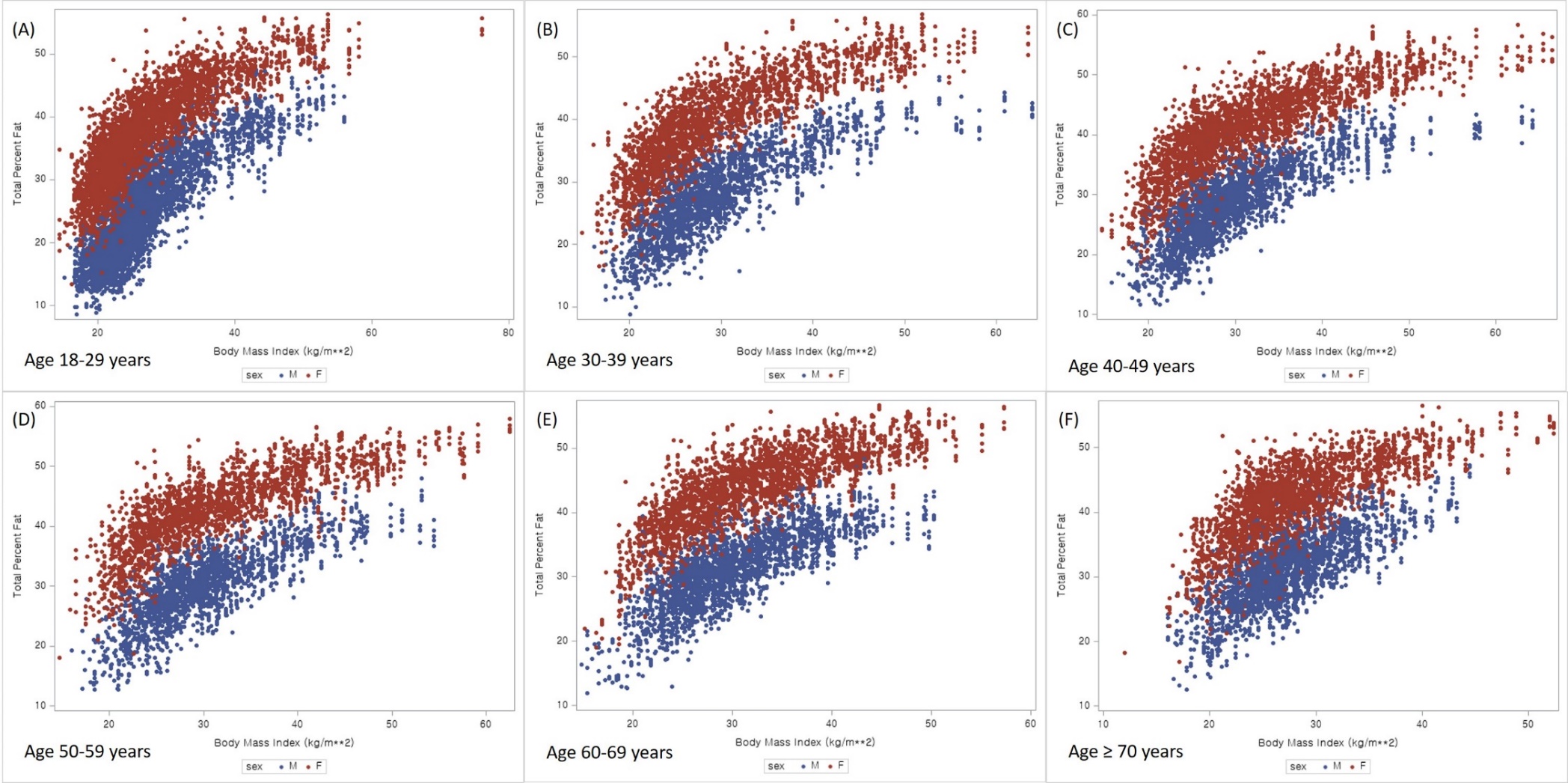
**

**Supplementary Figure 5**. Scatter plots for body mass index and fat mass index by race-ethnicity and sex

Legend: The plots of A - D show relationships between body mass index and fat mass index (FMI) in (A) whites (B) blacks (C) Mexican-Americans, and (D) Asians (Korean).


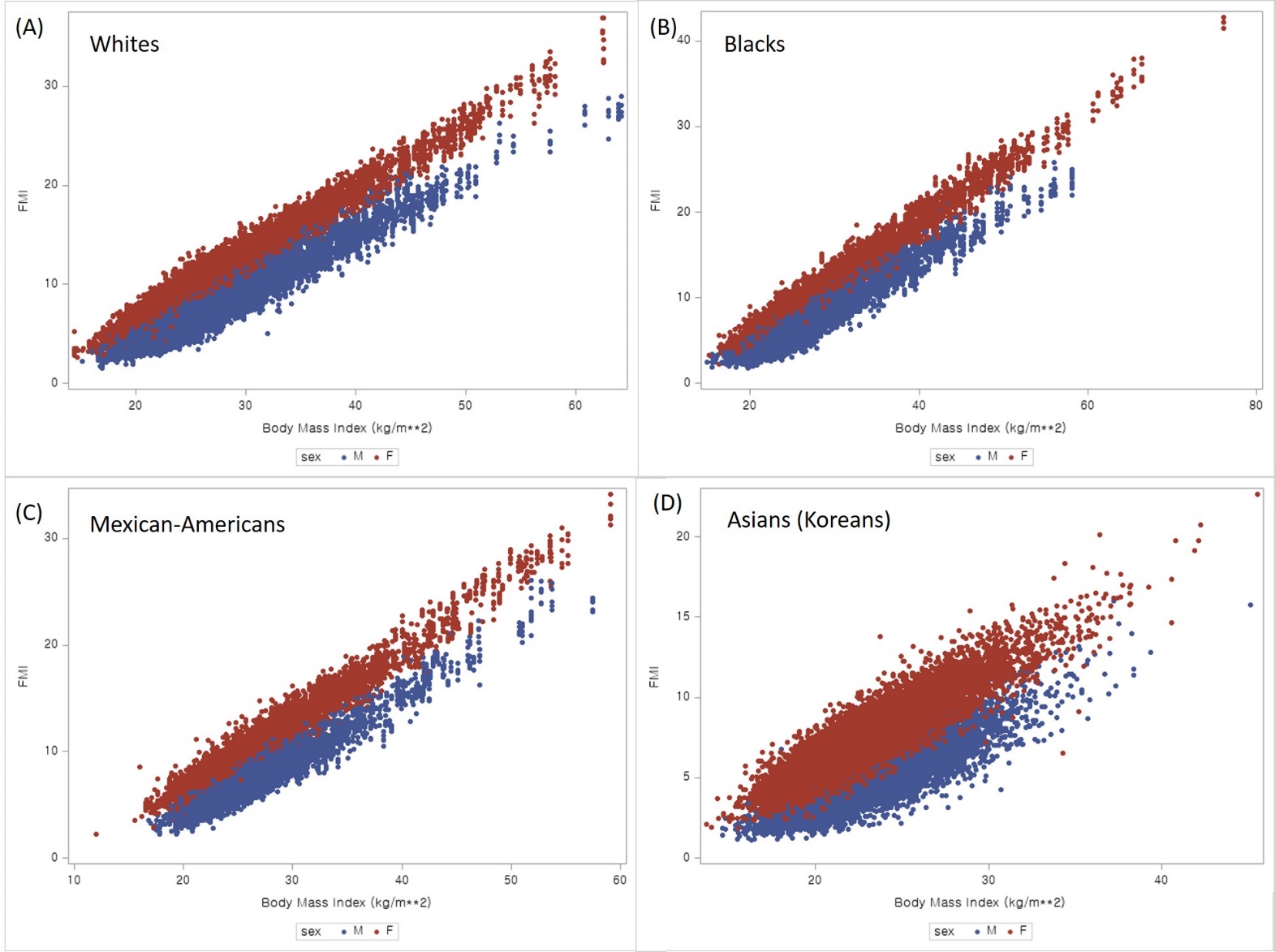


**Supplementary Figure 6.** Scatter plots for body mass index and percentage body fat by race-ethnicity and sex

Legend: The plots of A - D show relationships between body mass index and percentage body fat in (A) whites (B) blacks (C) Mexican-Americans, and (D) Asians (Korean).


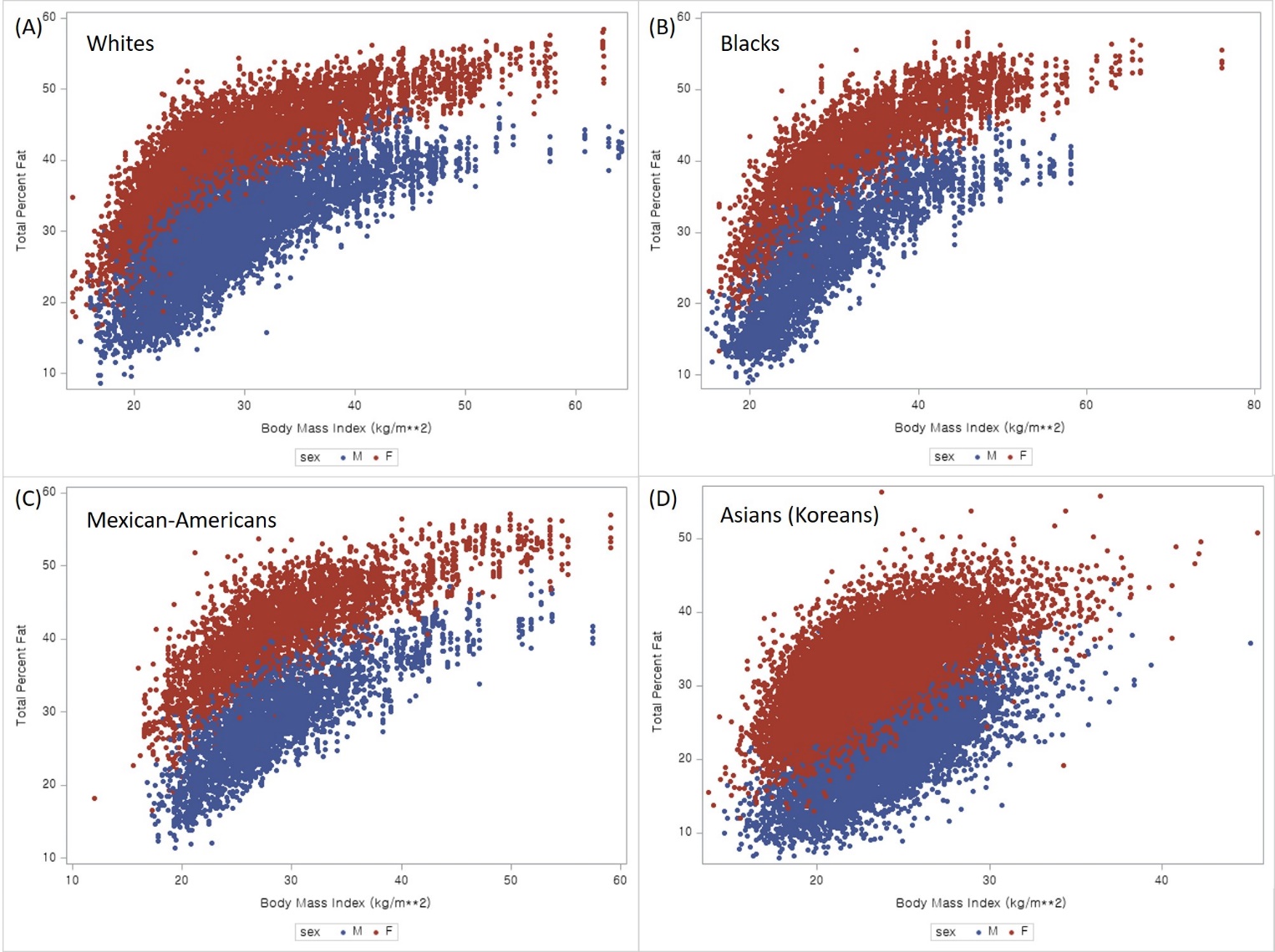


**Supplementary Figure 7**. Pearson correlation coefficients between obesity-related biomarkers and abdominal obesity indices (waist circumference and truncal fat mass) by sex and age

Legend: The bar graphs of panel A show coefficients between obesity-related biomarkers (total cholesterol, low-density lipoprotein cholesterol [LDL-C], high-density lipoprotein cholesterol [HDL-C], triglycerides [TG], C-reactive protein [CRP], hemoglobinA1c [HbA1c], fasting blood glucose [FBG] and insulin) and body mass index (BMI), fat mass index (FMI), and percentage body fat (PBF) in National Health and Nutrition Examination Survey (NHANES) and panel B shows the correlations in Korea NHANES. CRP was not available in Korea NHANES.


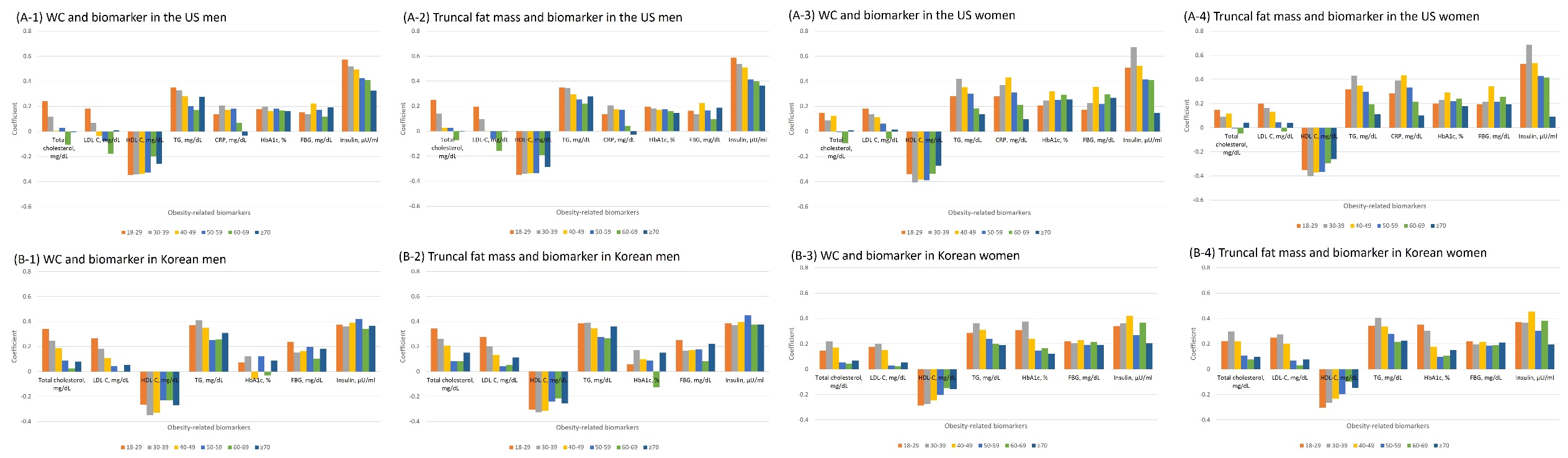


**Supplementary Figure 8**. Area under the curve (AUC) for identifying insulin resistance by body mass index, waist-height ratio (WHtR), waist circumference, fat mass index, and percentage body fat in the US population and Korean

abbreviation: National Health and Nutrition Examination Survey [NHANES], Korea NHANES [KNHANES]


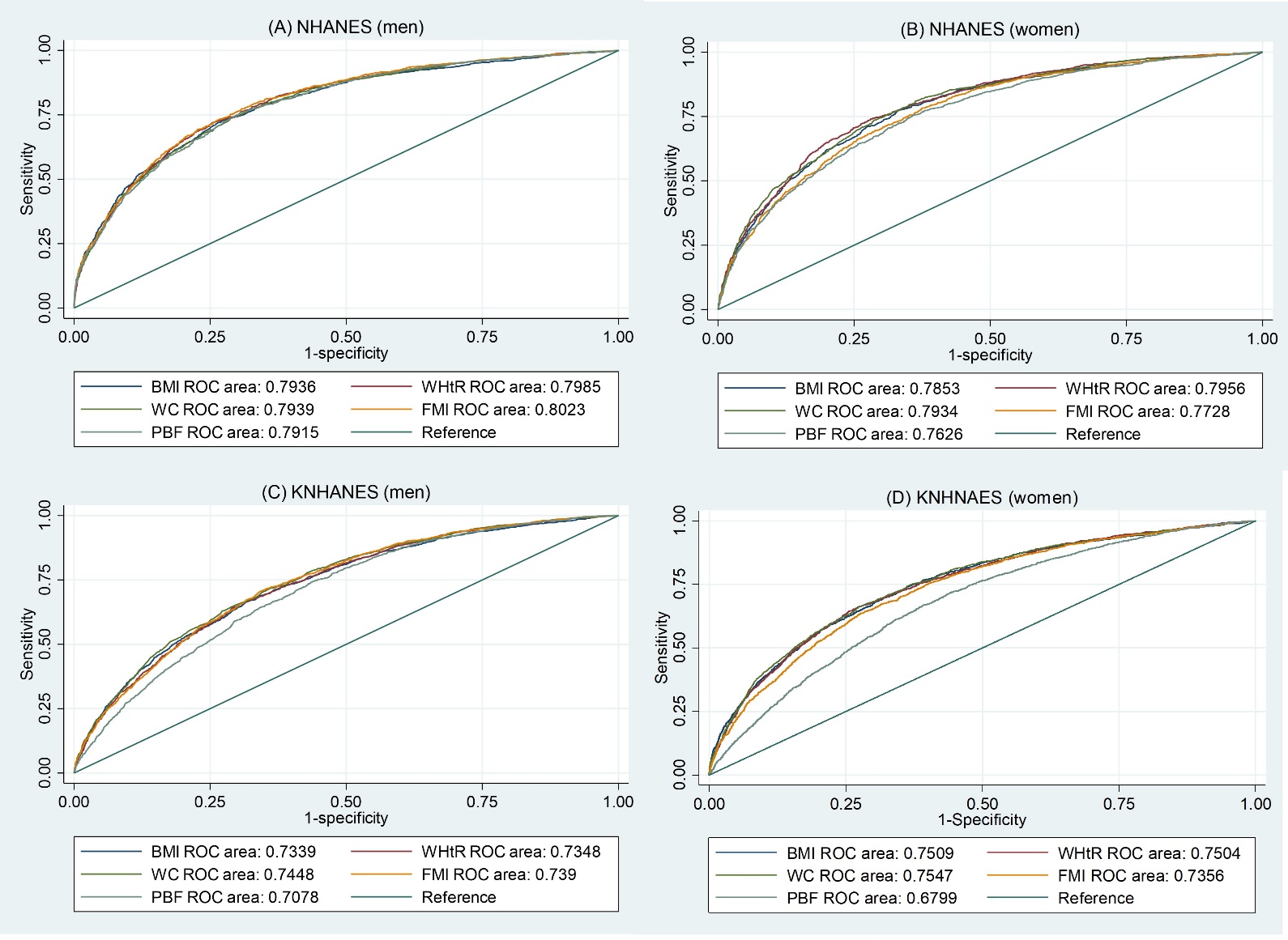


**Supplementary Figure 9**. Scatter plots for body mass index and fat mass index by sex in Korean

Legend: The plots of A - F show relationships between body mass index and fat mass index in (A) 18-29 years (B) 30-39 years (C) 40-49 years, (D) 50-59 years (E) 60-69 years, and (F) ≥ 70 years.


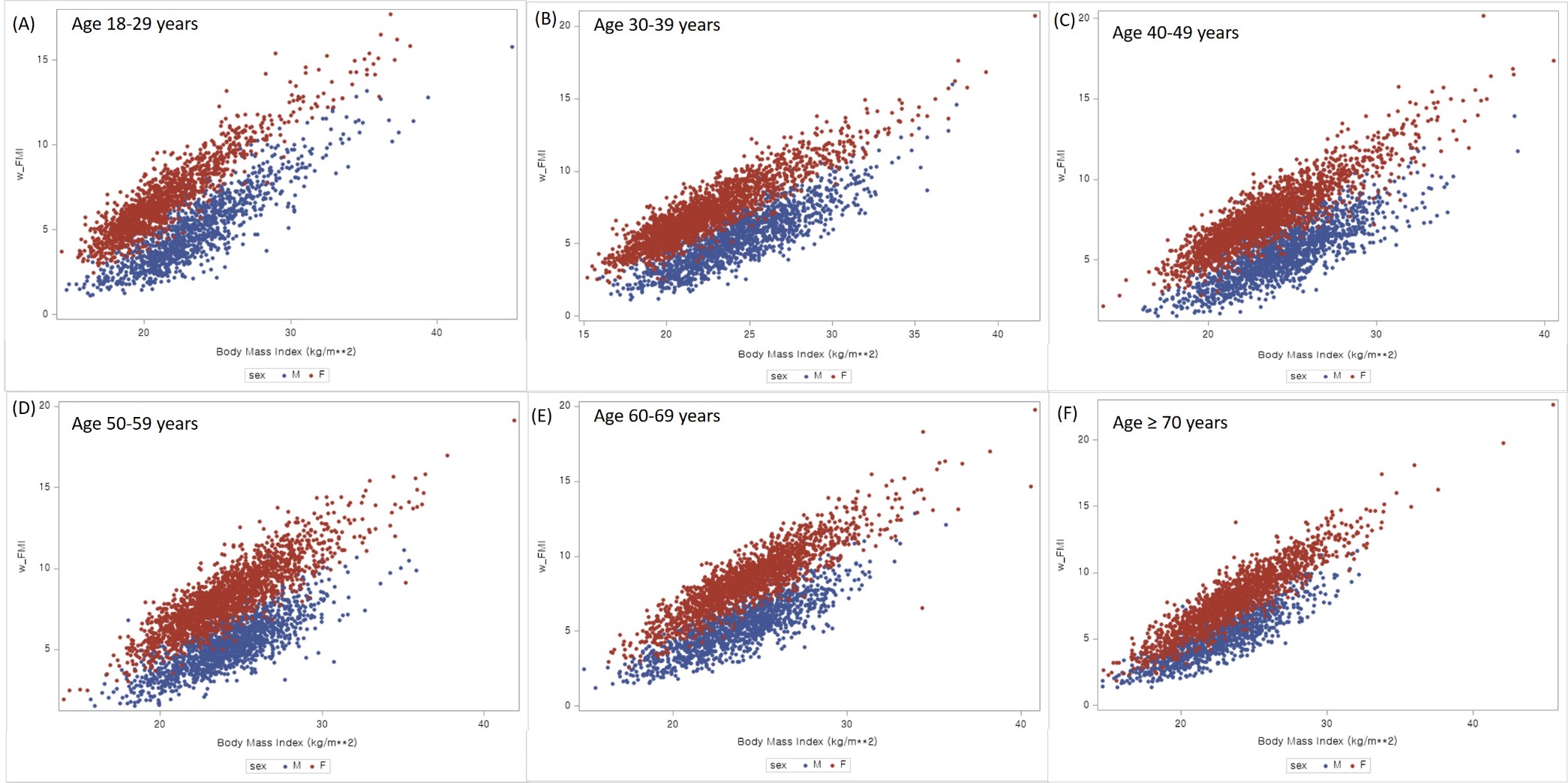


**Supplementary Figure 10.** Scatter plots for body mass index and percentage body fat by sex in Korean

Legend: The plots of A - F show relationships between body mass index and percentage body fat in (A) 18-29 years (B) 30-39 years (C) 40-49 years, (D) 50-59 years (E) 60-69 years, and (F) ≥ 70 years.


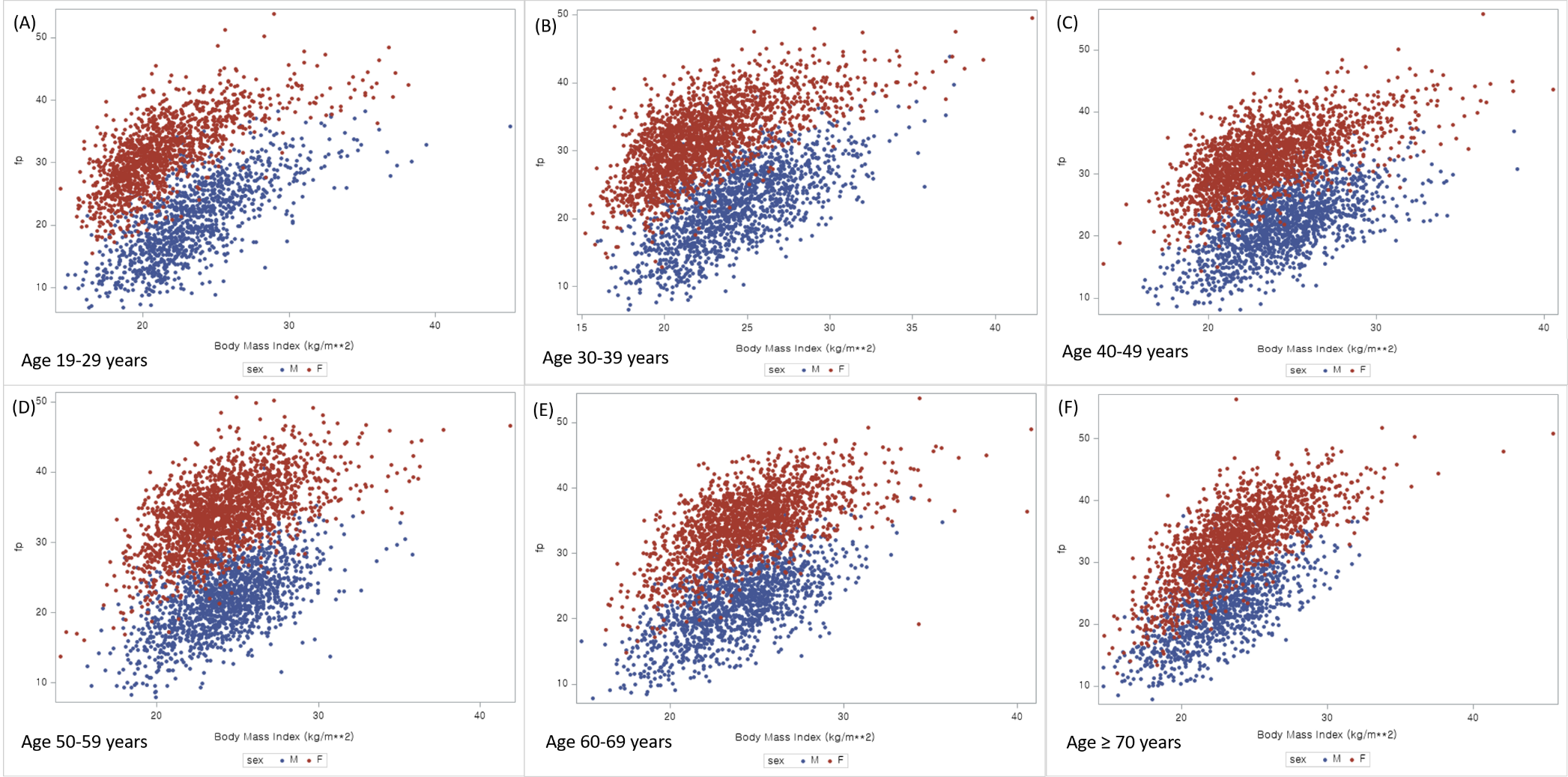

Supplement: Supplementary file 1 — Supplementary Information. [file 41598_2023_30527_MOESM1_ESM.docx]
